# Exendin-4 ameliorates tau hyperphosphorylation and cognitive impairment in type 2 diabetes through acting on Wnt/β-catenin/NeuroD1 pathway

**DOI:** 10.1186/s10020-023-00718-2

**Published:** 2023-09-04

**Authors:** Xiaonan Kang, Dan Wang, Lu Zhang, Teng Huang, Siyue Liu, Xiaohui Feng, Yaoyao Guo, Ziyin Zhang, Zhongjing Wang, Huihui Ren, Gang Yuan

**Affiliations:** 1 Department of Endocrinology, Tongji Hospital, Tongji Medical College, Huazhong University of Science and Technology, Wuhan, 430030 China; 2 Branch of National Clinical Research Center for Metabolic Disease, Hubei, People’s Republic of China; 3 Department of Endocrinology, The Central Hospital of Wuhan, Tongji Medical College, Huazhong University of Science and Technology, Wuhan, People’s Republic of China

**Keywords:** Type 2 diabetes, Cognitive impairment, Tau hyperphosphorylation, Glucagon-like peptide-1 receptor agonist, Wnt/β-catenin pathway, NeuroD1

## Abstract

**Background:**

Type 2 diabetes (T2D) is an independent risk factor for Alzheimer's disease (AD). Exendin-4 (Ex-4), a widely used glucagon-like peptide-1 receptor agonist drug in the treatment of T2D, has been demonstrated the therapeutic effects on diabetic encephalopathy (DE). Especially, the Ex-4 ameliorates the tau hyperphosphorylation and cognitive impairment in DE. And these crucial alterations are also important bridge between T2D and AD. However, its unique mechanism is unclear.

**Methods:**

The db/db mice, high-fat-diet (HFD) / streptozotocin (STZ)—induced diabetic (HF-diabetic) mice, and high-glucose-damaged (HGD) HT-22 hippocampal cells were enrolled to examine the effects of Ex-4 on AD-like changes in T2D. The Novel object recognition test (NORT) and Morris water maze test (MWMT) were conducted to evaluate the cognitive impairment. The Dickkopf-1 (DKK1) was employed to weaken the activation of the Wnt/β-catenin pathway to explore the mechanism of Ex-4 in protecting the brain functions. The JASPAR was based to predict the interaction between NeuroD1 and the promoter region of *Ins2*. Moreover, the chromatin immunoprecipitation coupled with quantitative polymerase chain reaction (ChIP-qPCR) and luciferase reporter assays were performed.

**Results:**

Ex-4 alleviated the tau hyperphosphorylation, increased the brain-derived insulin, and improved the PI3K/AKT/GSK3-β signalling in db/db mice, HF-diabetic mice, and HGD HT-22 hippocampal neuronal cells. The NORT and MWMT indicated that Ex-4 alleviated the learning and memory deficits in HF-diabetic mice. The inhibitor Dickkopf-1 (DKK1) of the Wnt/β-catenin pathway significantly blocked the protective effects of Ex-4. Regarding further molecular mechanisms, NeuroD1 was affected by Ex-4 in vivo and in vitro, and the knockdown or overexpression of NeuroD1 suggested its crucial role in promoting the brain insulin by Ex-4. Meanwhile, the ChIP‒qPCR and luciferase reporter assays confirmed the combination between NeuroD1 and the promoter region of the insulin-encoding gene *Ins2*. And this interaction could be promoted by Ex-4.

**Conclusions:**

Our study proposes that Ex-4 alleviates tau hyperphosphorylation and cognitive dysfunction by increasing *Ins2*-derived brain insulin through the Wnt/β-catenin/NeuroD1 signaling in T2D. And its also show new lights on part of the progress and mechanism on treatment targets for the DE in T2D.

**Supplementary Information:**

The online version contains supplementary material available at 10.1186/s10020-023-00718-2.

## Introduction

Metabolic diseases often cause systemic metabolic disorders, unbalancing the homeostasis and damaging various organs and tissues. Type 2 diabetes (T2D), a chronic metabolic disease characterized by glucose metabolism disorder and insulin resistance, has become an important healthcare burden in China and worldwide (Magliano and Boyko 2021). Brain damage in diabetes is named diabetic encephalopathy (DE), which is described as a cognitive dysfunction caused directly or indirectly by metabolic disorders, including hyperglycaemia, insulin resistance, and insulin deficiency (Duarte 2015). Recent studies have shown a certain connection between Alzheimer's disease (AD) and T2D. AD has even been considered the brain-specific type 3 diabetes. The β-amyloid (Aβ) and hyperphosphorylated tau are the main pathological hallmarks of AD, and the latter is considered more important as it can be detected before the Aβ deposition in the early pathophysiological stage (Boutajangout and Wisniewski 2014). Notably, tau phosphorylation is upregulated in the diabetic brain (Ramos-Rodriguez et al. 2013; Verdile et al. 2015). In addition, the metabolic disorder represented by hyperglycaemia and insulin resistance could promote the development of AD-like changes in T2D (Ma et al. 2015; Ramos-Rodriguez et al. 2013). There is neither an effective treatment for DE nor satisfaction efforts to control the corresponding symptoms currently (Thota et al. 2020). Therefore, new strategies for early prevention and treatment of AD-like changes in T2D have become the key to reversing the DE development into AD.

Exendin-4 (Ex-4), a glucagon-like peptide-1 (GLP-1) analogue in treating T2D, is highly affinitive and specifically actives the GLP-1 receptor (GLP-1R) (Fehmann et al. 1994; Schepp et al. 1994). GLP-1R is highly expressed in regions where the central nervous systems are, including the hippocampus and hypothalamus (Holst 2007; Shimizu et al. 1987). Although there is some controversy in humans, most rodent studies suggest that Ex-4 can cross the blood-brain barrier (BBB) and affects the brain (Kastin and Akerstrom 2003). In reality, Ex-4 efficiently protects nerve cells and PC12 cells against injury induced by Aβ or high glucose (Chen et al. 2012; Li et al. 2010). Besides, some scholars have found that the elevated brain insulin is crucial in treating the AD-like changes in T2D, and Ex-4 reduces the AD-like tau hyperphosphorylation in T2D by promoting the brain insulin and insulin signalling (Reger et al. 2006; Yang et al. 2013). In general, insulin in brain is mainly derived from peripheral blood via BBB. However, very little peripheral insulin can access the brain, because its capability of passing the BBB is greatly weakened by hyperinsulinemia and brain insulin resistance in T2D through the selective and saturated transport of capillary endothelial cells (Arnold et al. 2018; El Khoury et al. 2014; Freude et al. 2005). Therefore, effects of Ex-4 on promoting the brain-derived insulin and reducing the tau hyperphosphorylation in T2D are crucial for the treatment of DE and preventing DE from developing into AD. However, the involving mechanisms are largely unknown.

The Wnt/β-catenin pathway in the brain is related to the development and differentiation of the nervous system, the occurrence and development of AD, and the central insulin production (Gui et al. 2013; Yang et al. 2016). Studies have shown that directly activating or inhibiting the key negative regulators of the Wnt/β-catenin pathway can improve cognitive impairment in AD (Wan et al. 2014). Ex-4 can stimulate the cAMP-PKA pathway by activating GLP-1R and then increases the level of nonphospho-β-catenin (np-β-catenin) to stimulate the Wnt/β-catenin pathway (Liu and Habener 2008). And The production of brain-derived insulin can be detected after the activation of the Wnt pathway in the hypothalamus by Wnt3a (Lee et al. 2016).

Therefore, in this work, we employed db/db mice and established the high-fat-diet (HFD) / streptozotocin (STZ) -induced diabetic (HF-diabetic) mice and high-glucose-damaged (HGD) neuron cell model to evaluate the definitely therapeutic effects of Ex-4, and explored the underlying mechanisms which may closely related with DE, bran-derived insulin and Wnt/β-catenin signaling pathway.

## Research design and methods

### Animal groups and drug treatment

The 8-week-old male C57BL/6 mice and diabetic db/db (C57BLKS/J-LepRdb/LepRdb) mice were purchased from the HFK Bioscience Co., Ltd. (HFK) (China). All mice were maintained at room temperature (25 ± 2 °C) under a standard 12-h/12-h light–dark cycle with free access to water and food. All operation procedures related to the animals were approved by the Animal Care Committee of the Tongji Hospital, Huazhong University of Science and Technology. And complied with the standards described in the National Institutes of Health Guidelines for the Care and Use of Laboratory Animals.

Sixty male C57BL/6 mice were randomly assigned to the HFD/STZ induced T2D mice (HF-diabetic mice, n = 50) and the control mice (CTL mice, n = 10). They were fed a HFD (D12492; Research Diets; containing 60 kcal% fat, 20 kcal% carbohydrate, and 20 kcal% protein; from HFK.) or a normal control diet (NCD) (containing 10 kcal% fat, 70 kcal% carbohydrate, and 20 kcal% protein; from HFK.). After 12 weeks feeding, the mice fed with HFD were injected intraperitoneally with STZ (Sigma, USA) at 30 mg/kg for three consecutive days, while the CTL mice were injected with the sodium citrate buffer. Weight and the whole blood glucose (WBG) were monitored 3 days after the last injection and once a week thereafter. Mice with the random WBG levels higher than 16.7 mmol/L were set for the experimental T2D, and the others were excluded from this work. Finally, the CTL mice were regarded as the CTL group (subcutaneous injection and intranasal saline). The HF-diabetic mice were randomly divided into five groups, including the HF-diabetic group (subcutaneous injection and intranasal saline), the Ex-4 group (subcutaneous injection of Ex-4 and intranasal saline), the Ex-4 + DKK1 group (subcutaneous injection of Ex-4 and intranasal DKK1), the insulin-S group (subcutaneous injection of insulin and intranasal saline), and the insulin-I group (subcutaneous injection of saline and intranasal insulin).

Twenty-five male db/db mice were fed a NCD and randomly assigned to 5 groups (n = 5). The groups included the db/db group (subcutaneous injection and intranasal saline), the Ex-4 group (subcutaneous injection of Ex-4 and intranasal saline), Ex-4 + DKK1 group (subcutaneous injection of Ex-4 and intranasal DKK1), Insulin-S group (subcutaneous injection of insulin and intranasal saline), and Insulin-I group (subcutaneous injection of saline and intranasal insulin). Weight and WBG were measured weekly during the administration.

Specifically, each mouse was treated daily with both subcutaneous injection and nasal drip for 4 weeks. 2 IU insulin (Yang et al. 2013) (Novolin R, Novo Nordisk, China) or 3 mg/kg DKK1(Wei et al. 2018) (MCE, China) in 20 μl saline or equal saline was applied to intranasal drug delivery to the mice in the insulin-I group and the Ex-4 + DKK1 group by an Eppendorf pipette. The insulin at 6.7 IU/kg, approximately equivalent to 2 IU/mouse (Yang et al. 2013), Ex-4 at 10 μl /kg (Peng et al. 2021; Yang et al. 2016), or equal saline was subcutaneously injected into the mice in the insulin-S group, Ex-4 group, DKK1 + Ex-4 group, and other groups. The Insulin-I group was set as the positive control due to its certain effect on alleviating tau hyperphosphorylation and cognitive impairment, and the Insulin-S group was defined as the WBG control to explore the changes by lowering the WBG only. All administrations were applied simultaneously each day. Finally, all mice were sacrificed for further studies after assessment of the cognitive behaviour.

### Assessment of the cognitive behaviour

#### Novel Object Recognition test (NORT)

The NORT was employed to evaluate the rodents' instinct to explore novel objects, which determined their recognition and memory abilities according to the experimental methods previously described (Liu et al. 2020). The time and frequency of exploration of exploring the objects were measured. The recognition ability was analysed by comparing the time or numbers spent exploring the two same objects or the novel object in the test.

#### Morris Water Maze test (MWMT)

The MWM test was performed to evaluate the spatial learning and memory ability (Liu et al. 2020). Moreover, the single probe test was performed on the second day after the last train. The time to reach the platform (escape latency), distance swam to the platform, swam speed, and the time and distance spent in each quadrant, were measured and recorded by the video tracking system.

### Measurements of insulin and C-peptide in mouse cerebrospinal fluid (CSF)

After continuous light anaesthetization with 0.5% pentobarbital, 6–8 μl of mouse CSF was collected from the cisterna magna and stored in Eppendorf vials (Ma et al. 2015). After quick freezing in liquid nitrogen, the CSF was stored at -80 °C. Levels of insulin and c-peptide in the CSF were measured by the enzyme-linked immunosorbent assay (EZ assay, China).

### Tissue preparation

After the mice were decapitated, the brains were rapidly removed and sagittal cut into the left and right hemispheres on an ice-cooled board. Next, the right hemisphere was fixed in 4% paraformaldehyde to prepare the routine paraffin Sects. (4 μm thick) for morphological studies. Besides, the hippocampus was dissected from the left hemisphere and stored at -80 °C for biochemical analyses.

### Cell culture, treatment, and transfection

The mouse hippocampal neuronal cell line HT22 and human embryonic kidney (HEK) 293 T cells were purchased from the American Type Culture Collection (ATCC Virginia, USA). And they were cultured in the Dulbecco's modified eagle medium (DMEM) basic (Gibco, 10569044, USA) supplemented with 10% foetal bovine serum (Gibco, 16140071, USA), 100 IU/ml penicillin, and 100 μg/ml streptomycin. Cells maintained in a 37 °C incubator with 5% CO_2_ and grown to 70–90% confluence were collected for analyses unless otherwise noted.

To establish an in vitro model of chronic high glucose damage and explore the effect of Ex-4. Because the optimal growth and survival rate of HT-22 cells requires 25 mM basic glucose, high glucose DMEM containing 25 mM glucose was used as the control glucose (CON) group. An additional 25 mM glucose was added to DMEM (total of 50 mM) as the high glucose (HG) group (Chen et al. 2019; Jingxuan et al. 2022). Mannitol (25 mM) was added to the DMEM (HM) group as control to eliminate the influence by osmotic pressure in the HG group. HT22 cells were incubated in high-glucose (50 mM) DMEM for 48 h and then treated with Ex-4 (10 nM) for another 48 h (HG + Ex-4) (Hattori et al. 2000; Peng et al. 2021).

The *Ins2*-knockdown HT22 cells (V-*Ins*2) were constructed by lentivirus containing short hairpin RNA (shRNA) targeting *Ins2* (target sequence 5’-3’: GCAGGTGACCTTCAGACCTTG, designed by RIBOBIO, China). Then, they were co-cultured with its negative control (V-CON) at 50 mM for 48 h. Next, they were again cultured with or without the Ex-4 (HG + Ex-4 or HG) for another 48 h to determine whether the effects of Ex-4 on HT22 cells were attributed to increased insulin synthesis due to *Ins2* transcription.

The Wnt/β-catenin signaling inhibitor was treated as follows. The HT22 cells under high glucose (50 mM) were treated with DMSO (HG) or Ex-4 (10 nM, HG + Ex-4) or 100 ng/μl DKK1 for 2 h (Wang et al. 2017), and then added with Ex-4 (10 nM, HG + DKK1 + Ex-4) or without Ex-4 (HG + DKK1) to react another 48 h.

NeuroD1 overexpressed (NeuroD1-KI), NeuroD1 knockdown (NeuroD1-KD), and negative control (NC) HT22 cells were constructed by the lentivirus transfection to clarify the role of NeuroD1 when the Ex-4 affected the HGD HT22 cells. Lentivirus production and transient transfections were performed by Lipofectamine 3000 (Thermo Fisher Scientific, USA) according to the manufacturer’s instructions. The target sequence 5’-3’ for the lentivirus containing shRNA targeting *NeuroD1* is GACAACATTATGTCTTTCGAT. All plasmids were produced by the Designgene Biotechnology Co. Ltd (China).

### Western blotting

Hippocampus or HT22 cells were homogenized in radioimmunoprecipitation assay (RIPA) buffer (MedChemExpress, USA) containing complete protease inhibitor cocktail and phosphatase inhibitor cocktail I and II (MedChemExpress, USA). Western blotting was performed as previously described (Peng et al. 2021). The involved primary antibodies were listed in Additional file 1: Table S1.

### Quantitative real-time polymerase chain reaction (RT‒PCR) analysis

The cells were subjected to the quantitative RT-PCR with SYBR Green PCR Master Mix (Vazyme, China) and analysed using a Roche RT-PCR System as previously described (Peng et al. 2021). The primer sequences are given in Additional file 1: Table S2.

### Immunohistochemistry (IHC) staining and immunofluorescence (IF) staining

The hippocampus was isolated intact and fixed with ice-cold 4% paraformaldehyde (PFA) for 24 h, and the brain was dehydrated in 30% sucrose-PBS for 48 h. According to a previous method (Wang et al. 2021), the IHC against tau (Thr231) (Abcam, UK) at 1:150 and IF against insulin (Abcam, UK) at 1:50 were performed. The images were captured using a microscope or automatic section scanning system (Olympus, Japan).

HT22 cells were seeded on poly-L-lysine–coated coverslips. According to a previous method (Peng et al. 2021), IF against insulin (CST, USA) at 1:50, β-catenin (CST, USA) at 1:200, and NeuroD1 (CST, USA) at 1:200 was performed. Each slide was observed with a fluorescence microscope (Olympus, Japan).

### Chromatin immunoprecipitation and chromatin immunoprecipitation quantitative PCR assay

HT22 cells from the CON group, HG group and HG + Ex-4 group were subjected to the Simple ChIP® Plus Sonication Chromatin IP Kit #56,383 (CST, USA). The chromatin was incubated overnight at 4 °C with the anti-NeuroD1 antibody (CST, USA), and a normal immunoglobulin G (IgG) antibody (CST, USA) was selected as the NC. The RT-PCR analysis were performed as described previously, and the primers were designed by Sangon Biotech Co., Ltd. (China).

### Transient transfection and luciferase reporter assay

During transfection of HT22 cells with the Lipofectamine 3000, the overexpressed plasmid (or vector) of the NeuroD1 was transfected with the pGL3-*Ins2*-WT, pGL3-*Ins2*-MUT1, and pGL3-*Ins2*-MUT2 luciferase reporters, respectively. *Ins2* transcriptional activity was assessed using the Dual Luciferase Report Assay Kit DL101-01(Vazyme, China) on a TD-20/20 Luminometer (Turner Bio Systems, USA).

### Statistical analysis

All the experiments above were performed in triplicate independently at least. The GraphPad Prism (version 8.3.0) was employed for statistical analysis, and all data were presented as means ± standard deviations (M ± SD). A two-tailed Student’s t test was applied to compare the data of the two groups with a normal distribution, and the one-way ANOVA with Tukey’s post hoc analysis was employed for the multiple comparison. The data difference was significant when *P* < 0.05.

### Data and resource availability

The datasets, and adenoviruses and plasmids are available by a reasonable request from the corresponding authors.

## Results

### Ex-4 alleviated tau hyperphosphorylation in the hippocampus of T2D models

**Fig. 1:**
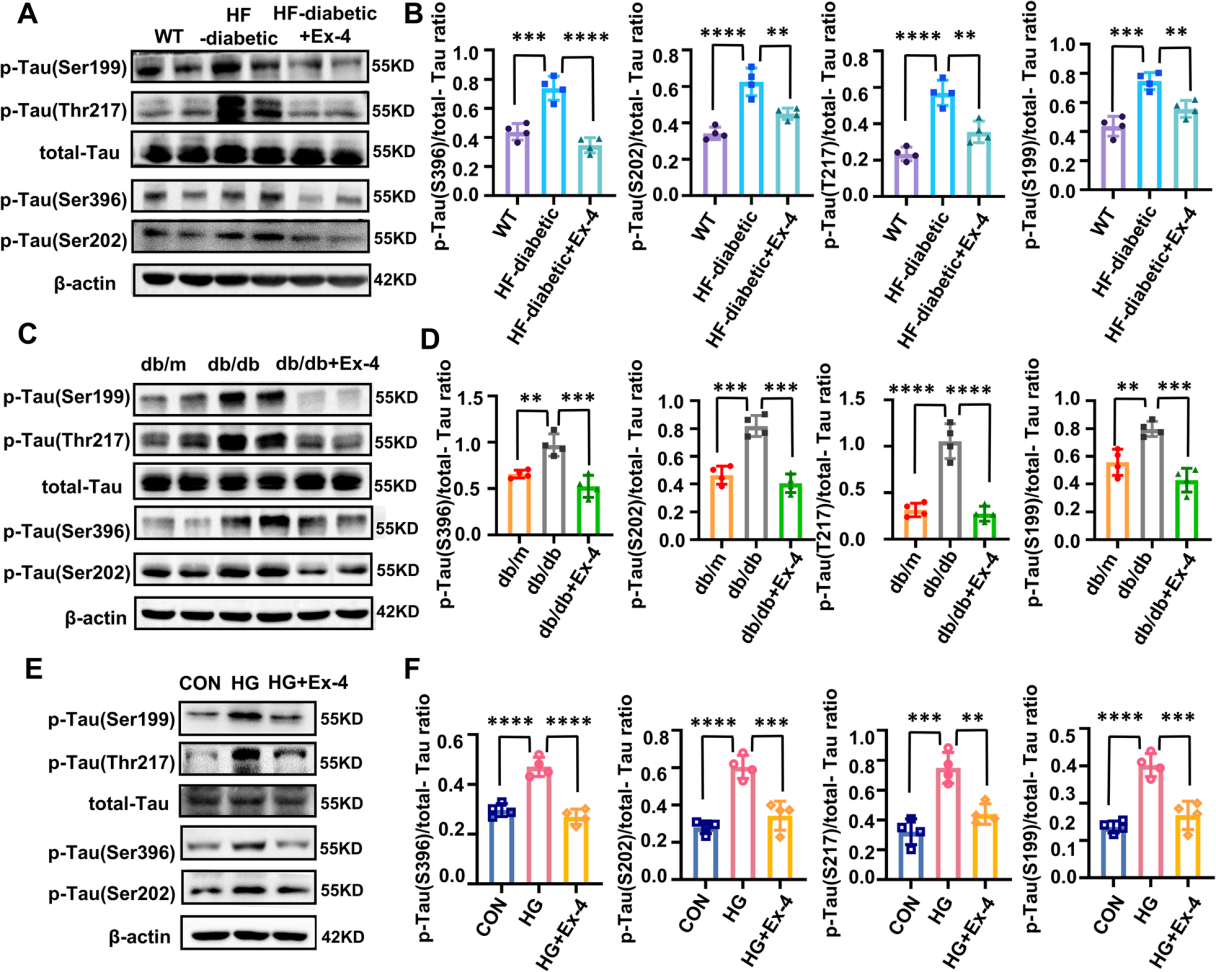
Ex-4 ameliorates tau hyperphosphorylation in the T2D mice hippocampus and high-glucose-injured HT22 cells. **A** Male C57BL/6 mice were divided into three groups. Mice fed a normal diet as the WT group; others establish the HF-diabetic mice model. The HF-diabetic mice which treated daily with exendin-4 (10 µg/kg) for 4 weeks as the Ex-4 group. The phosphorylated tau at Ser199, Ser202, Ser396, and Thr217 sites and total tau in the hippocampus were examined by immunoblotting. **B** Gray density of A. **C** Male db/db mice were divided into two groups. One was treated daily with exendin-4 (10 µg/kg) for 4 weeks, and the others were treated with saline, db/m mice as the control. The phosphorylated tau at Ser199, Ser202, Ser396, and Thr217 sites and total tau in the hippocampus were examined by immunoblotting. **D** Gray density of C. **E** HT22 cells under the control (CON) or high glucose (50 mM, HG) environment for 48 h, then part of cells in the HG group treated with exendin-4 (10 nM, HG + Ex-4) for 48 h. Immunoblot demonstrates changes in phosphorylated tau at Ser199, Ser202, Ser396, and Thr217 sites and total tau. **F** Gray density of E. β-actin was used as the internal standard. For **A**, **C**, and **E**, n = 4/group. All results are representative of three independent experiments. Values are presented as mean ± SD. ** P < 0.01, *** P < 0.001, **** P < 0.0001

Tau hyperphosphorylation of several AD-associated has been reported in the brains of T2D patients (Liu et al. 2009) and rodents (El Khoury et al. 2014). The most sites with tau hyperphosphorylation in AD, including Ser199, Ser202, Ser396, and Thr217, were detected in this work. The HF-diabetic mice (Fig. 1A, B) and the db/db mice (Fig. 1C, D) both exhibited an increased phosphorylated tau at the above four sites compared to their normal control group. On the other hand, mice in the db/db + Ex-4 and HF diabetic + Ex-4 groups presented a greatly reduced level of phosphorylated tau. In addition, a similar phenomenon was observed in HT22 cells (Fig. 1E, F). The HT22 cells demonstrated an increased level of tau hyperphosphorylation at these four sites in the HG group compared with those in the CON group, but a great reduced level in the HG + Ex-4 group than the HG group. It should be noticed that the osmotic pressure influences were excluded (Additional file 1: Fig. S1).

### 3.2 Ex-4 promoted the *Ins2*-induced brain-derived insulin production and insulin signaling activation to decrease the level of tau hyperphosphorylation through the Wnt/β-catenin pathway in T2D mice

To investigate the origin of the Ex-4-induced brain insulin in T2D and explore the role of the involved Wnt/β-catenin pathway, experiments were carried out in two independent T2D mouse models, including the HF-diabetic mice and db/db mice.

**Fig. 2:**
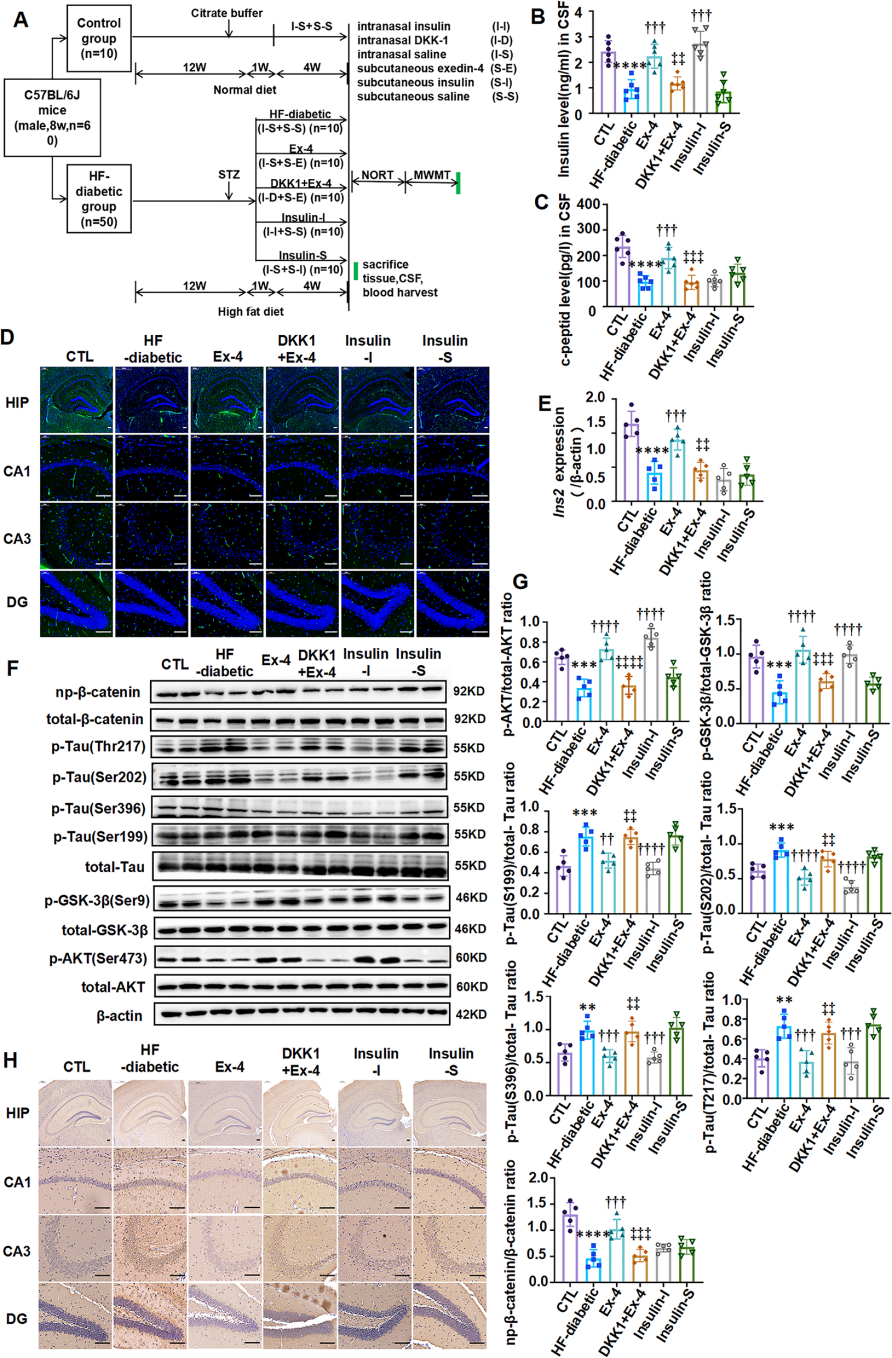
Ex-4 reduces tau hyperphosphorylation by increasing brain-derived insulin via activating Wnt/β-catenin pathway in HF-diabetic mice. **A** Workflow of the animal study for C57BL/6 mice. Insulin levels **B** and c-peptide levels **C** in CSF were measured by ELISA kits (n = 6/group). **D** Represented immunofluorescence staining coronal brain sections of each group mice were performed for insulin (green) and DAPI (blue). Scale bar = 100 μm. **E** mRNA levels of *Ins2* in the hippocampus were measured by RT-qPCR assay. β-actin as the internal control. (n = 5/group). **F** The levels of the phosphorylated tau at Ser199, Ser202, Ser396, and Thr217 sites and total tau in each group; the activation of Wnt/β-catenin pathway as evidenced by the levels of the np-β-catenin to total β-catenin; insulin signalling activation as P-AKT^S473^ to total AKT and P-GSK-3β^S9^ to total GSK-3β were examined through Western blot analysis, (n = 5/group). **G** The gray density of E. **H** Represented immunohistochemical staining of coronal brain sections from mice of each group was performed with the antibody against phosphorylated tau at Thr231. Scale bar = 100 μm. For **D** and **H**, HIP: hippocampus, CA: cornue ammonis, DG: dentate gyrus. Results are representative of three independent experiments. Values are presented as mean ± SD. **P < 0.01, ***P < 0.001, ****P < 0.0001. vs. CTL; ^††^P < 0.01, ^†††^P < 0.001, ^††††^P < 0.0001. vs. HF-diabetic; ^‡‡^P < 0.01, ^‡‡‡^P < 0.001, ^‡‡‡‡^P < 0.0001. vs. Ex-4

Figure 2A described the experimental workflow in the HF-diabetic mice. After four weeks of daily administration, the weight and WBG of mice in the HF-diabetic group were much higher than those in the CTL group but effectively decreased in the Ex-4 group and DKK1 + Ex-4 group. In the Insulin-S group, although hyperglycaemia was ameliorated, body weight was increased. Besides, the increased WBG and weight in the Insulin-I group were slight (Additional file 1: Fig. S2A–D). Meanwhile, the levels of insulin (Fig. 2B) and c-peptide (Fig. 2C) in CSF, insulin in the hippocampus (Fig. 2D), and the transcription of the insulin-encoding gene *Ins2* (Fig. 2E) were all markedly lower in the HF-diabetic group than in the CTL group, but all higher in the Ex-4 group than those in the HF-diabetic group. Additionally, the phosphorylation of Ser473-protein kinase B (AKT) and Ser9-glycogen synthase kinase 3 beta (GSK-3β) was more obvious and the tau hyperphosphorylation in hippocampus was decreased more greatly in the Ex-4 group than the HF-diabetic group. Therefore, activation of the Wnt/β-catenin pathway was greatly weakened by reducing the nonphospho-β-catenin (np-β-catenin) to total β-catenin under the action of DKK1 in the DKK1 + Ex-4 group (Fig. 2F–H). Besides, such changes in the Insulin-S group were slight compared with those in the HF-diabetic group. In addition, the Insulin-I group took increased insulin level and phosphorylation of Ser473-AKT and Ser9-GSK-3β in CSF but reduced levels of tau hyperphosphorylation and c-peptide (Fig. 2B–H).

**Fig. 3:**
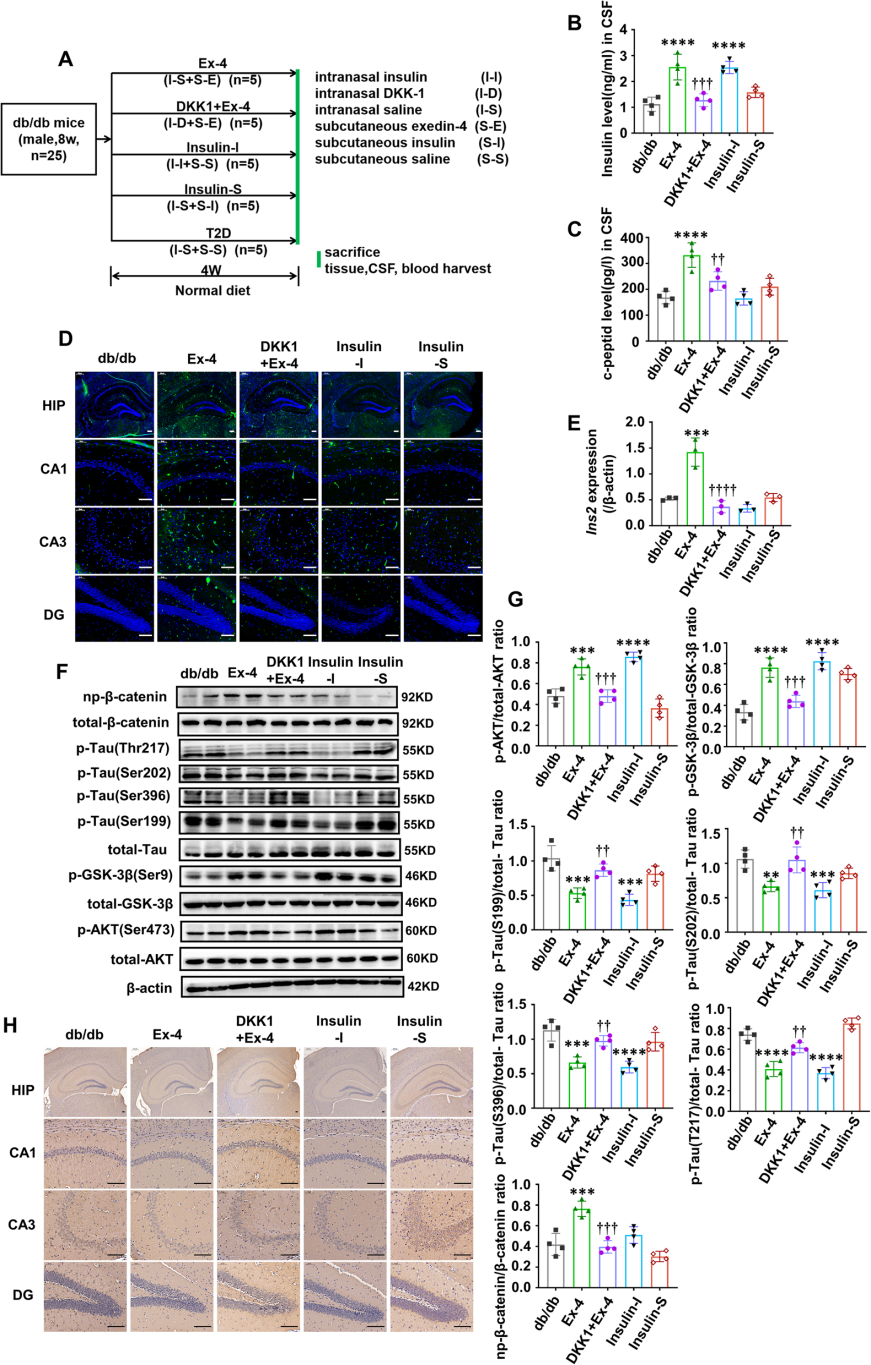
Ex-4 promotes brain-derived insulin by activating Wnt/β-catenin pathway to antagonize tau hyperphosphorylation in db/db mice. **A** Workflow of animal study designed for db/db mice. Insulin levels **B** and c-peptide levels **C** in CSF were measured by ELISA kits (n = 4/ group). **D** Represented immunofluorescence staining of coronal brain sections obtained from mice of each group was performed for insulin (green) and DAPI (blue). Scale bar = 100 μm. **E** mRNA levels of *Ins2* in the hippocampus were measured by RT-qPCR assay. β-actin was the internal control. n = 3/group. **F** The levels of the phosphorylated tau at Ser199, Ser202, Ser396 and Thr217 sites and total tau in each group; the activation of Wnt/β-catenin pathway as evidenced by the levels of the np-β-catenin to total β-catenin; insulin signalling activation as P-AKT^S473^ to total AKT and P-GSK-3β^S9^ to total GSK-3β were examined through Western blot analysis, (n = 4/group). **G** The gray density of E. **H** Represented immunohistochemical staining of coronal brain sections from each group of mice was performed with the antibody against phosphorylated tau at Thr231. For **D** and **H**, HIP: hippocampus, CA: cornue ammonis, DG: dentate gyrus. Results are representative of three independent experiments. Values are presented as mean ± SD. **P < 0.01, ***P < 0.001, ****P < 0.0001. vs. db/db; ^††^P < 0.01, ^†††^P < 0.001. vs. Ex-4

Similar experiments were conducted in db/db mice to further validate the above findings. Figure 3A described the experimental workflow. The mice in the db/db group were regarded as the disease control to evaluate the effects of each administration, which demonstrated the same results as in the HF-diabetic mice. Compared with the db/db group, db/db mice in the Ex-4 group exhibited the elevated levels of insulin and c-peptide (Fig. 3B, C) in CSF, more insulin contains in the hippocampus (Fig. 3D) and enhanced *Ins2* transcription (Fig. 3E), more phosphorylation of Ser473-AKT and Ser9-GSK-3β, and decreased tau hyperphosphorylation (Fig. 3F, G). However, all these changes were extremely weakened by intranasal dropping DKK1 to inhibit the activation of Wnt/β-catenin pathway (Fig. 3B–H). Furthermore, the mice in the Insulin-I group showed a lower tau phosphorylation but an increased insulin generation and an enhanced insulin signaling activation (Fig. 3B–H). In the contrast, the Insulin-S group showed very little changes in these factors (Fig. 3B–H). The changes of WBG and weight were consistent with those in the HF-diabetic mice (Additional file 1: Fig. S2E, F).

### Ex-4 alleviated the injured cognitive and memory function in T2D mice via the Wnt/β-catenin pathway

Behavioural experiments, including the NORT and MWZT, were conducted to evaluate cognitive and memory abilities.

**Fig. 4:**
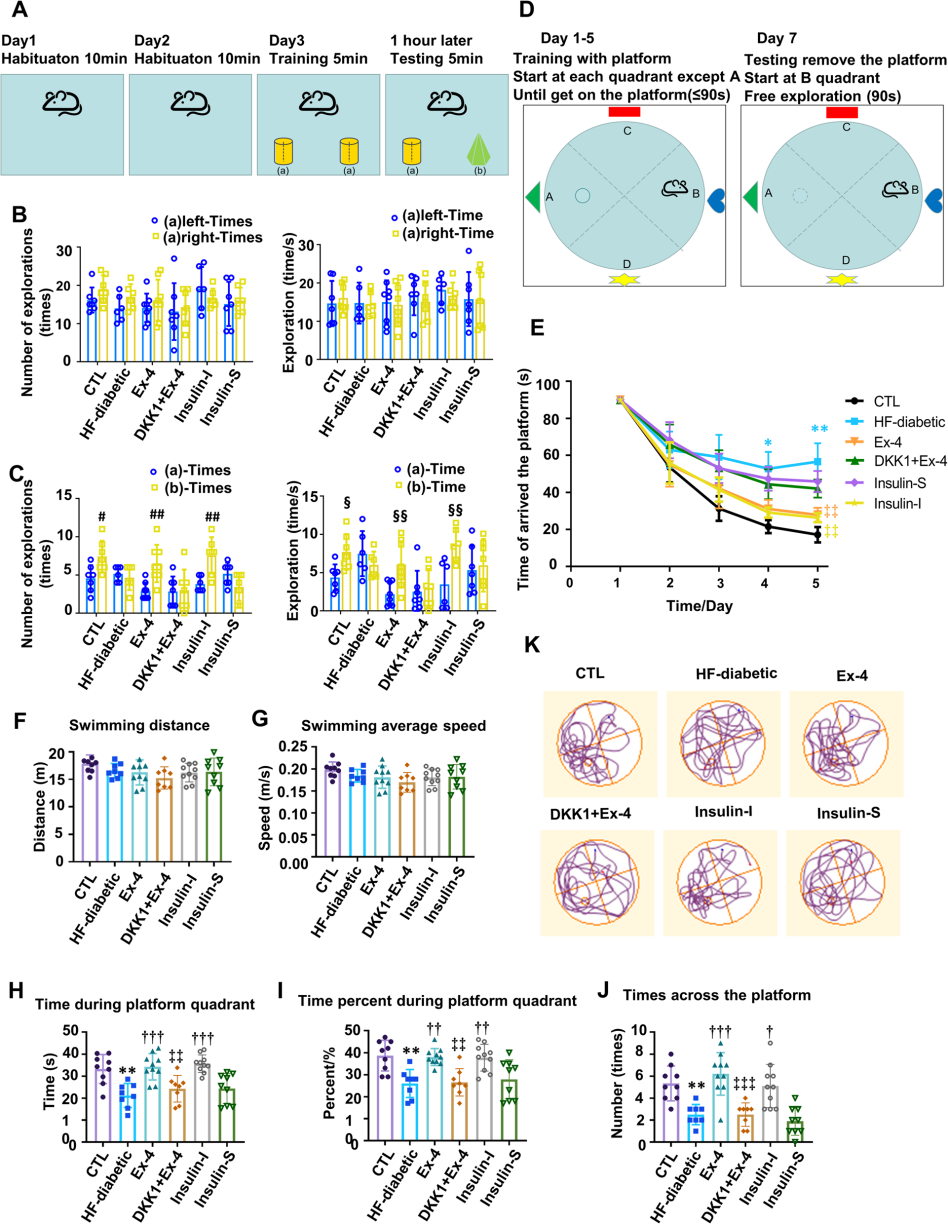
Ex-4 alleviates cognitive impairments in T2D mice by activating the Wnt/β-catenin signalling in the hippocampus. **A**–**C** Results of the novel object recognition test (NORT), **D**–**J** results of Morris water maze test (MWMT). **A** Schematic diagram of the NORT. **B** The number and time of the mice explored the same two objects in the familiarity stage. **C** The number and time of the mice explored the two different objects in the new object recognition stage (n = 7/group). **D** A schematic diagram of the MWMT **E** Represented latency during the acquisition phase. **F** Total swam distance during the test day. **G** Average swam speed during the test day. **H** Time spent by mice in targeted quadrant during the test day **I** Represented the percent of time spent by mice in the targeted quadrant and the average of other quadrants. **J** Total numbers of crossing over the platform during the test day. (K) Represented video tracks of probe trials on the test day. For **E**–**J** n = 8–10/group. Values are presented as means ± SD. For C, **A** ^#^P < 0.05, ^##^P < 0.01. for the time spent on two objects in each group; and ^§^ < 0.05, ^§§^P < 0.01. for the explored numbers spent on two objects in each group. For F-J, **P < 0.01, ***P < 0.001.vs CTL; ^†^P < 0.05, ^††^P < 0.01, ^†††^P < 0.001. vs. HF-diabetic; ^‡‡^P < 0.01, ^‡‡‡^P < 0.001. vs. Ex-4

Figure 4A showed the protocol of the NORT. Two identical objects were presented on the training day, while one familiar object and a novel object were placed on the testing day. The results illustrated that the location preference was not greatly different among various groups on the training day (Fig. 4B). The time and frequency for exploring new objects were significantly lower than those for old objects in the HF-diabetic group than those in the CTL group. Besides, both the time and frequency increased in the Ex-4 group and the Insulin-I group but changed slightly in the DKK1 + Ex-4 group and Insulin-S group (Fig. 4C).

The schematic diagram of the MWMT was illustrated in Fig. 4D. The learning curve of mice in each group was displayed in Fig. 4E. It can be observed that the spatial learning and memory abilities of mice in the HF-diabetic group were obviously weaker than those in the CTL group, while the Insulin-I group and Ex-4 group showed the reversed results. However, the beneficial effect of Ex-4 was weakened in the DKK1 + Ex-4 group. In addition, the Insulin-S group and the HF-diabetic group showed no great difference in the learning ability (Fig. 4E). The motor performance of all mice appeared normal because the swimming speed and total distance were exactly same (Fig. 4F, G). The time spent in the target quadrant (Fig. 4H), the percent time spent in the target quadrant (Fig. 4I), the number of crossing the platform areas (Fig. 4J), and the movement tracks on the probe trial (Fig. 4K) were evaluated on the testing day. In detail, the mice in the HF-diabetic group spent less time and touched fewer numbers of crossing the platform areas in the target quadrant, demonstrating a diminished MWMT performance compared to those in the CTL group. Besides, the mice in the Insulin-I group and Ex-4 group had significantly better memory for the target than those in the HF-diabetic group. In contrast, the results in the Insulin-S group were little different from those in the HF-diabetic group. And the results in the Insulin-S group were little different from those in the DKK1 + Ex-4 group. Such results suggested that Ex-4 could alleviate the cognitive memory impairment via the Wnt/β-catenin pathway in the T2D model mice.

### Ex-4 decreased the tau hyperphosphorylation by *Ins2*-induced insulin production in HT22 cells

**Fig. 5:**
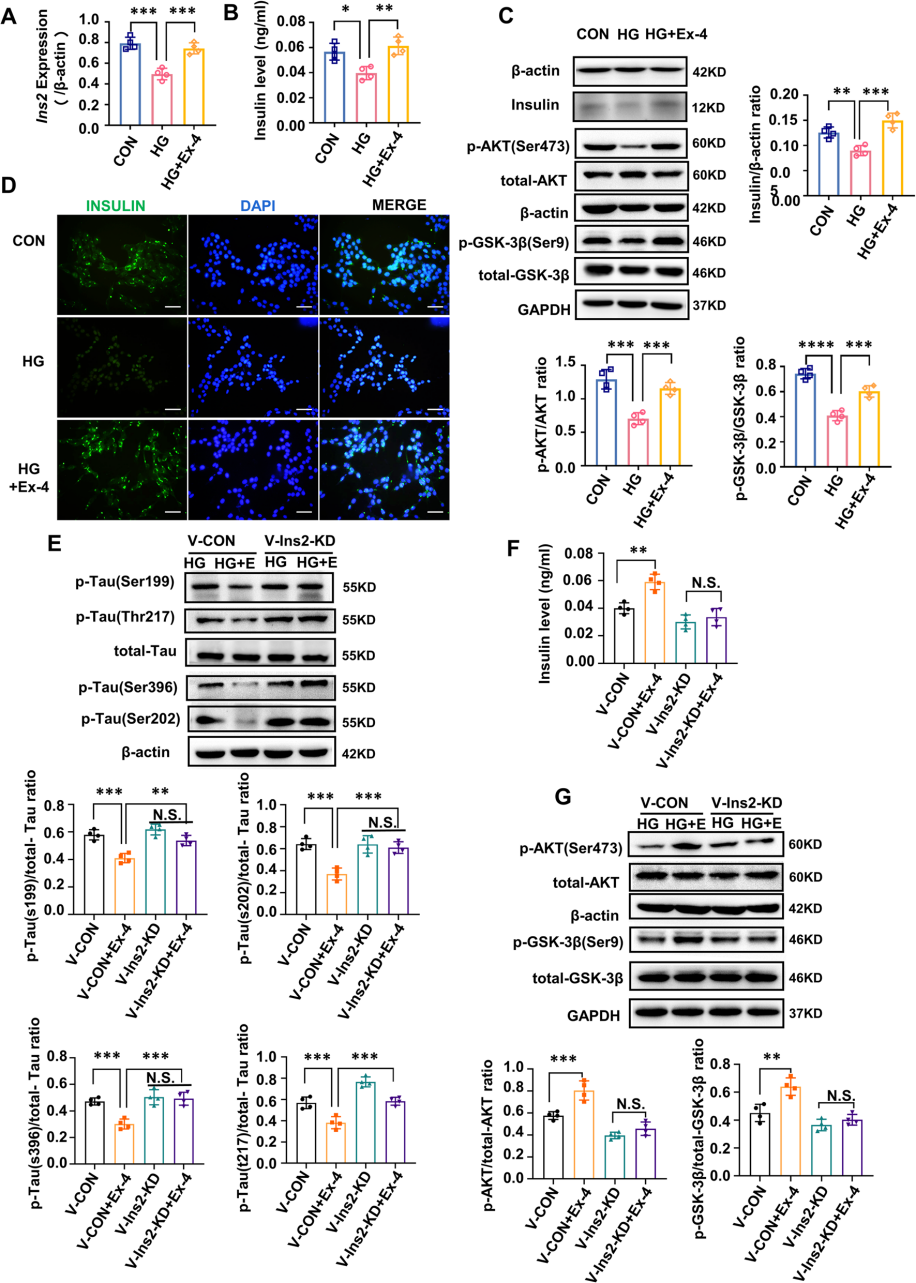
Ex-4 increases the *Ins2*-encoding insulin to reduce the tau hyperphosphorylation in the high-glucose-damaged HT22 cells. **A**–**D** HT22 cells under the control (CON) or high glucose (50 mM, HG) environment for 48 h, then part of cells in HG group treated with exendin-4 (10 nM, HG + Ex-4) for 48 h. **A** mRNA levels of *Ins2* in groups were measured by RT-qPCR assay. β-actin was the internal control. n = 4. **B** The insulin levels of the culture supernatant in groups were detected by an ELISA kit. n = 4. **C** The insulin production and insulin signalling activation as P-AKT^S473^ to total AKT and P-GSK-3β^S9^ to total GSK-3β were examined through Western blot analysis and their gray density. n = 4. **D** Represented immunofluorescence staining of HT22 cells was performed for insulin including proinsulin (green) and DAPI (blue). Scale bar = 100 μm. **E**–**G** HT22 cells were cultured in high glucose (50 mM, HG) environment for 48 h after infection with *Ins2*-knockdown and its negative control lentivirus. Then, they were cultured in high glucose (50 mM, HG) environment with or without Ex-4 (10 nM) for another 48 h. **E** Immunoblot and gray density demonstrated the levels of phosphorylated tau at specific sites and total tau. n = 4. **F** Insulin levels in culture supernatant in groups measured by ELISA kit. n = 4. **G** Immunoblot and the gray density for insulin with insulin signalling factors, including total AKT, P-AKT^S473^, total GSK-3β, and P-GSK-3β^S9^. n = 4. Results are representative of three independent experiments. Values are presented as mean ± SD. *P < 0.05, **P < 0.01, ***P < 0.001, ****P < 0.0001; N.S. no significant difference

The levels of *Ins2* and insulin were downregulated in HT22 cells in the HG group compared with the CON group, which were partly reversed in the HG + Ex-4 group (Fig. 5A, B, D). In addition, the phosphorylation of Ser473-AKT and Ser9-GSK-3β were both decreased in the HG group, which was alleviated by Ex-4 (Fig. 5C). To further investigate whether such alleviation was related to the level of insulin and the activation of insulin signaling, the *Ins2* knockdown-expressing HT22 cells were constructed (Additional file 1: Fig. S3). The *Ins2* knockdown and normal HT22 cells were treated with Ex-4 under high-glucose medium. Ex-4 reduced the tau hyperphosphorylation slightly (Fig. 5E) and failed to up-regulate the level of insulin (Fig. 5F) or increase the phosphorylation of Ser473-AKT and Ser9-GSK-3β (Fig. 5G) in the *Ins2* knockdown cells, while its effects were obvious in normal HT22 cells. This supported that the elevated *Ins2*-induced brain insulin was important for Ex-4 to play its role.

### Ex-4 promoted the *Ins2*-induced insulin production and insulin signaling activation to alleviate the tau hyperphosphorylation through the Wnt/β-catenin pathway in HT22 cells

**Fig. 6:**
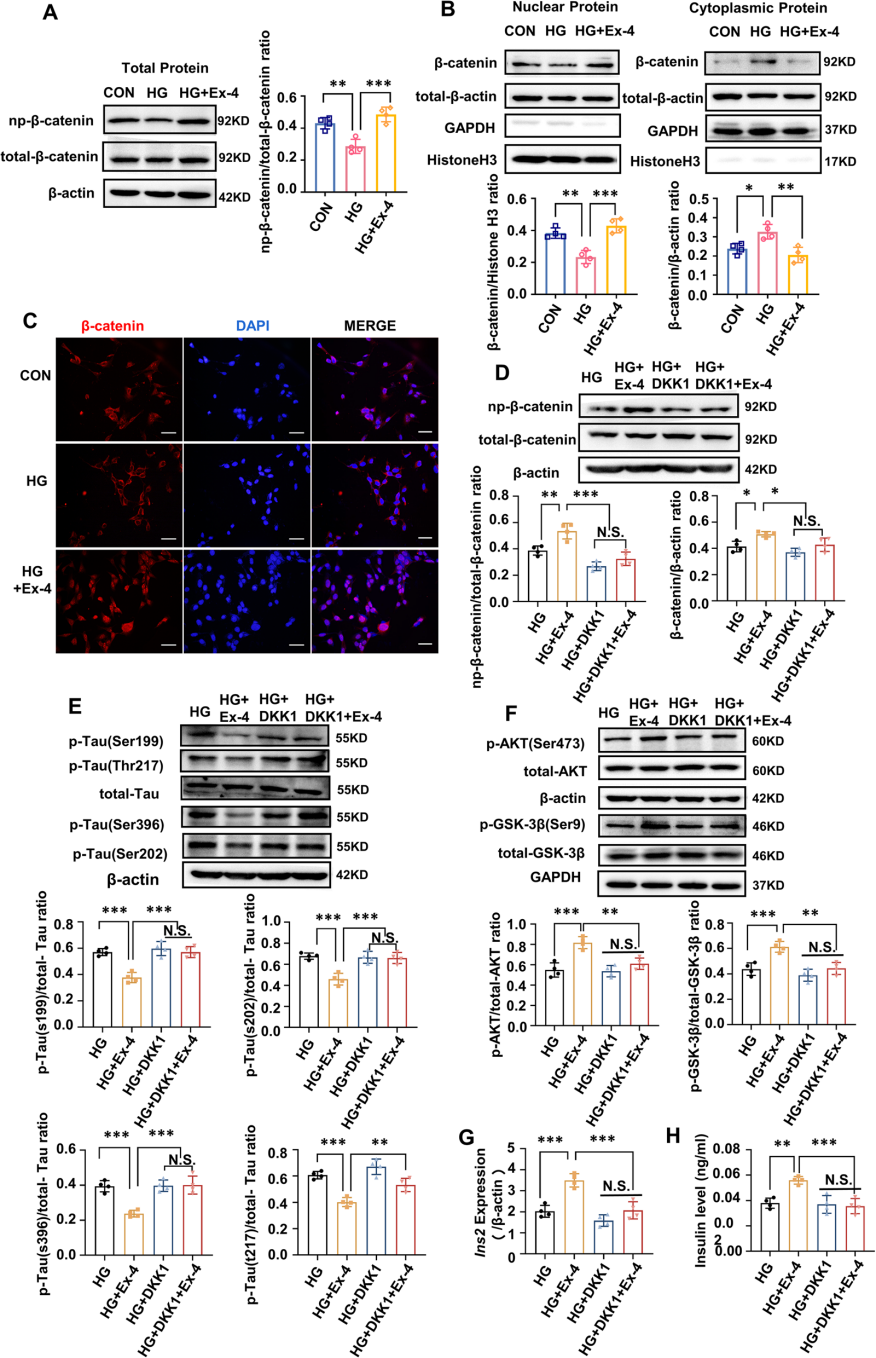
Ex-4 promotes *Ins2*-encoding insulin via the Wnt/β-catenin pathway in the high-glucose-damaged HT22 cells. **A**–**C** HT22 cells under the control (CON) or high glucose (50 mM, HG) environment for 48 h, then part of the cells in the HG group were treated with exendin-4 (10 nM, HG + Ex-4) for 48 h. **A** Immunoblot and its gray density demonstrated the level of np-β-catenin to total β-catenin; β-actin was the internal control. n = 4. **B** Nuclear and cytoplasmic fractions were separated to describe the level of β-catenin by immunoblotting and its gray density. Histone H3 and β-actin as internal control, respectively. n = 4. **C** Represented immunofluorescence for β-catenin (red) and DAPI (blue). Scale bar = 100 μm. **D**–**H** HT22 cells were cultured in high glucose (50 mM, HG) environment for 48 h and then divided into four groups. The HG group was treated with saline, and the HG + Ex-4 group was treated with 10 nM Ex-4, the HG + Dkk1 group was treated with 100 ng/μl Dkk1, the HG + DKK1 + Ex-4 group was pretreated with 100 ng/μl Dkk1 for 2 h before Ex-4 was added, then kept for 48 h. **D** Immunoblot demonstrated the Wnt/β-catenin signalling activation by the level of np-β-catenin and total β-catenin. **E** Tau phosphorylated levels at specific sites and total tau were examined by Immunoblot. **F** The insulin signalling activation described by the total AKT, P-AKT^S473^, total GSK-3β, and P-GSK-3β^S9^ was examined through Immunoblot. n = 4. **G** mRNA levels of *Ins2* were measured by RT-qPCR assay. n = 4. (H) Insulin in the culture supernatants was measured by an ELISA kit. n = 4. For **D**–**G** β-actin as the internal standard. Results are representative of three independent experiments. Data are presented as the mean ± SD. *P < 0.05, **P < 0.01, ***P < 0.001; N.S. no significant difference

The effects of Wnt/β-catenin pathway on Ex-4 in HT22 cells were investigated. Meanwhile, activation of the Wnt/β-catenin signaling was discussed by analysing the np-β-catenin to total β-catenin in whole cell protein (Fig. 6A), and the β-catenin in isolated cytoplasmic protein and nuclear protein (Fig. 6B). It showed a decreased np-β-catenin to total β-catenin and a lower β-catenin in the nucleus in the HG group compared with the CON group. While such changes could be partly reversed in the HG + Ex-4 group. Moreover, the IF results indicated that the β-catenin expression was downregulated in the HG group compared with that in the CON group but was enhanced and translocated into the nucleus in the HG + Ex-4 group (Fig. 6C). DKK1 with and without Ex-4 administration was added to analyse whether the activation of Wnt/β-catenin signaling was indispensable for Ex-4 to alleviate the tau hyperphosphorylation (Fig. 6D). Furthermore, similar to the changes in the level of insulin, alleviation of the tau hyperphosphorylation by the Ex-4 was weakened by DKK1 (Fig. 6E). Additionally, the insulinotropic and increased phosphorylated Ser473-AKT and Ser9-GSK-3β affected by Ex-4 were attenuated by DKK1 (Fig. 6F), and the *Ins2* transcription (Fig. 6G) and insulin secretion (Fig. 6H) showed the similar results. It suggested that inhibiting the Wnt/β-catenin pathway attenuated the effects of Ex-4 on insulin secretion, PI3K/AKT/GSK-3β signaling activation, and the tau hyperphosphorylation of the HT22 cells.

### NeuroD1 played a crucial role in increasing the *Ins2*-induced brain-derived insulin secretion via the Wnt/β-catenin pathway

**Fig. 7:**
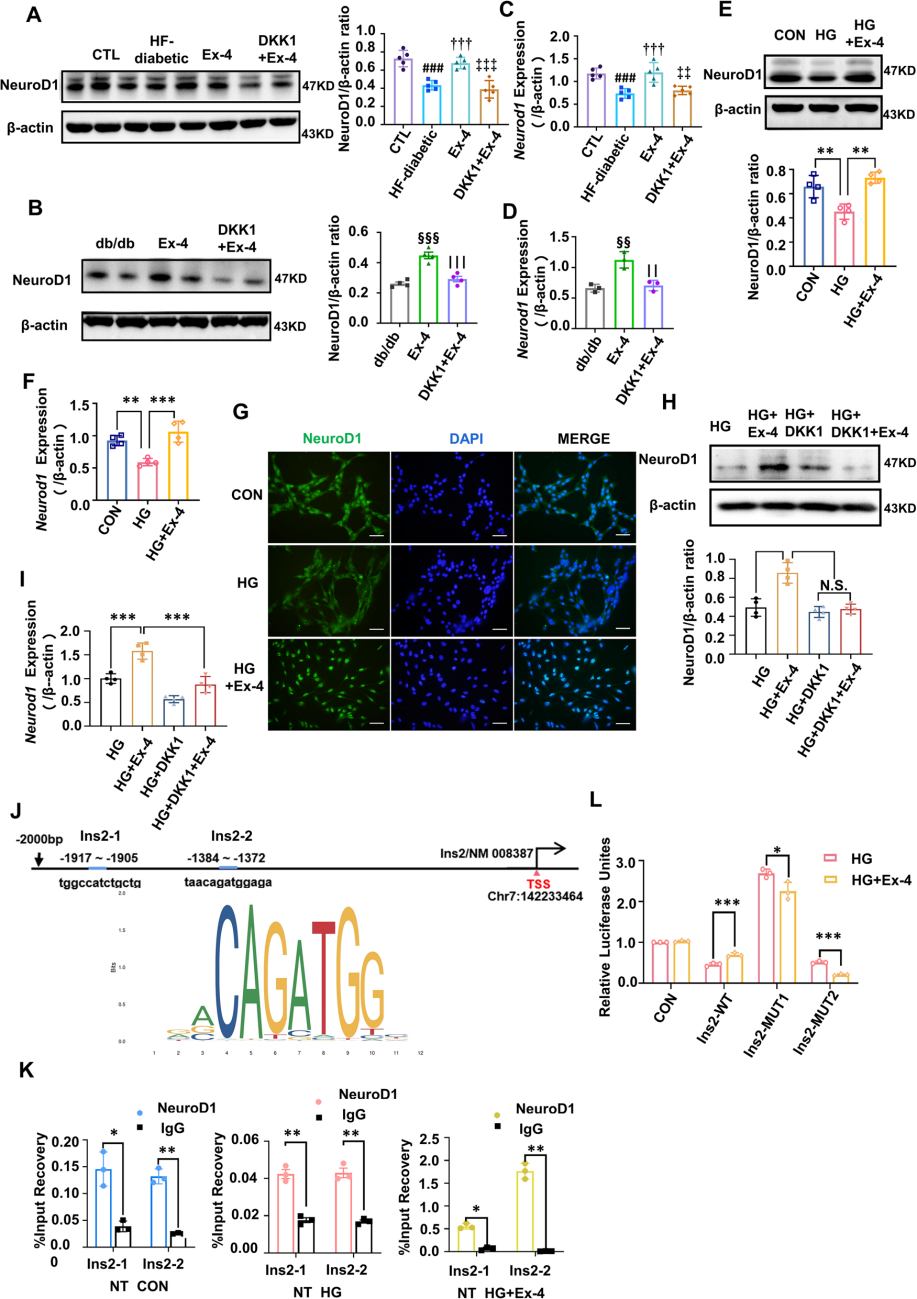
NeuroD1 is crucial on Ex-4 activating the Wnt/β-catenin signalling to promote the *Ins2*-encoding brain insulin. The protein levels of NeuroD1 in the hippocampus of HF-diabetic mice **A** and db/db mice **B** were detected by Western blotting. Additionally, the mRNA levels of *Neurod1* in the hippocampus of HF-diabetic mice **C** and db/db mice **D** were measured by RT-qPCR assay. **E**–**G** and **J**–**K** HT22 cells were treated as in Fig. 1C, And NeuroD1 level was demonstrated by immunoblot in (**E**). mRNA level of *Neurod1* **F** detected by RT-qPCR assay. **G** Immunofluorescence was performed for NeuroD1 (green) and DAPI (blue). Scale bar = 100 μm. **H** Immunoblot with its gray density and **I** mRNA levels measured by RT-qPCR demonstrated the NeuroD1 changes in HT22 cells treated as in Fig. 6D–H. **J** The binding sites of NeuroD1 on the *Ins2* promoter shown in the diagrammatic drawing, and the **K** ChIP-qPCR results of the relative binding levels of IgG or NeuroD1 to the input measured by the indicated primers are shown under the diagrammatic drawing. **L** HT22 cells were transfected with the NeuroD1 expression plasmid and the wild-type *Ins2* luciferase reporter construct or its mutation plasmids (MUT1/MUT2). Then, they were incubated in HG or HG + Ex-4 as above. Luciferase activity was measured and normalized to the Renilla control. β-actin was the internal control. Data are presented as the mean ± SD. For A and C, n = 5, ^###^P < 0.001. vs. CTL; ^††^P < 0.01, ^†††^P < 0.001. vs. HF-diabetic. ^‡‡^P < 0.001, ^‡‡‡^P < 0.001. vs. Ex-4. For B (n = 4) and D (n = 3), ^§§^P < 0.01, ^§§§§^P < 0.0001 vs. db/db; || P < 0.01. vs. Ex-4. And for **E**–**I** (n = 4), **J**–**K** (n = 3), *P < 0.05, **P < 0.01, ***P < 0.001

To explore the specific mechanism for the Ex-4-Wnt/β-catenin pathway-insulin production, changes in the messenger ribonucleic acid (mRNA) among those mainly insulin-related transcriptional regulators in the HT22 cells were detected under the high-glucose conditions with and without Ex-4. Meanwhile, the NeuroD1 was selected for the further study, as illustrated in Additional file 1: Fig. S4A. The protein and mRNA levels of NeuroD1 in the hippocampus were lowed in the T2D group than those in the control group, and such changes were rescued in the Ex-4 group. However, this effect of Ex-4 could be weakened by DKK1, as demonstrated in Fig. 7A–D. Similar results were also observed in HT22 cells (Fig. 7E, F). The IF results supported that NeuroD1 was decreased in the HG group HT22 cells and increased and shown nuclear localization in the HG + Ex-4 group (Fig. 7G). Additionally, the protein level (Fig. 7H) and transcription (Fig. 7I) of NeuroD1 were obviously inhibited by DKK1 no matter whether the Ex-4 was added, revealing that the effects of Ex-4 on NeuroD1 can be influenced by inhibiting the Wnt/β-catenin pathway.

Moreover, the NeuroD1-overexpressing HT22 cells (Additional file 1: Fig. S4B–D) showed more insulin and phosphorylated Ser473-AKT and Ser9-GSK-3β than the control cells under high-glucose conditions (Additional file 1: Fig. S4E). When the NeuroD1 was knocked down, the level of insulin, phosphorylation of Ser473-AKT and Ser9-GSK-3β, and the tau hyperphosphorylation exhibited little changes (Additional file 1: Fig. S4F), nor did those in the *Ins2* transcription (Additional file 1: Fig. S4G).

Besides, the possible binding sites of NeuroD1 on *Ins2* promoter regions were predicted by JASPAR for studying the mechanisms further, and two highest score sites were selected (Additional file 1: Table S3, Fig. 7J). The influences of Ex-4 on the binding between NeuroD1 and *Ins2* gene promoter were explored based on the ChIP-qPCR assay. The bindings of NeuroD1 on site 1 and site 2 were decreased by 35.6% and 50.6% in the HG group, respectively when they were compared with the bindings in the CON group. Meanwhile, such decrease can be reversed by a 3.53-fold and 73.17-fold increase at site 1 and site 2, respectively, after the Ex-4 treatment, in contrast to the HG group (Fig. 7K). Besides, a plasmid containing the wild-type (WT) *Ins2* promoter region, two mutant plasmids (MUT1 and MUT2) targeting the two binding sites, and a NeuroD1 overexpression plasmid were constructed, based on which the double luciferase experiment was carried out. Ex-4 treatment resulted in a 1.53-fold (WT-INS2), 0.84-fold (MUT1-INS2), and 0.40-fold (MUT2-INS2) in *Ins2* promoter activity compared with the HG group (Fig. 7L). It supported that NeuroD1 targeting the specific promoter regions of *Ins2* was crucial for the effects of Ex-4.

## Discussion

The mechanisms of DE has not been fully elucidated. Although it is already demonstrated that Ex-4 could alleviate the abnormal symptoms and pathological changes in DE of T2D, which were also described in this work (Figs. 1, 2, 3). However, its unique way of Ex-4 affect on DE is unknown. In this work, we conducted in vitro and in vivo molecular exploration and the behavioural experiments to elucidate the special mechanism of Ex-4 on alleviating the brain dysfunctions in T2D. The results indicated that the brain-derived insulin deficiency or the low-activation state of Wnt/β-catenin signaling may greatly contributed to DE. And more importantly, the Ex-4 alleviates the tau hyperphosphorylation and cognitive dysfunction by elevating the *Ins2*-derived brain insulin via Wnt/β-catenin/ NeuroD1 pathway in T2D.

Brain insulin exerts a crucial role in reducing the tau hyperphosphorylation in the hippocampus of T2D (Yang et al. 2013). The downregulated brain insulin in T2D weakens the activity of insulin signaling and over-activates the GSK-3β to hyperphosphorylated tau, contributing to occurrence and progression of AD (Noble et al. 2013; Yang et al. 2013). Intranasal insulin directly elevates brain insulin and significantly ameliorates the cognitive impairment in T2D patients (Gaddam et al. 2021), suggesting that enhancing the brain insulin is feasible in treatment of AD. However, very few peripheral insulin can access the brain through the BBB in T2D due to the hyperinsulinemia and brain insulin resistance (Arnold et al. 2018; El Khoury et al. 2014; Freude et al. 2005), which was also proven in our animal experiments, as subcutaneous insulin injection failed to remarkably increase the insulin level in CSF (Figs. 2B, 3B). In addition, the c-peptide level in CSF was also detected to reflect the changes in brain-derived insulin (Figs. 2C, 3C). The further exploration revealed that *Ins2* was crucial to lower the tau hyperphosphorylation in T2D and Ex-4 could not revert associated lesions in HGD HT22 cells after knockdown of the *Ins2* (Fig. 5). This indicates that Ex-4 ameliorates the tau hyperphosphorylation in T2D by promoting the *Ins2*-derived brain insulin in the hippocampus.

Furthermore, the mechanism of Ex-4 on promoting the *Ins2*-derived insulin in the hippocampus of T2D has not been clarified, which is the focus of this work. The Wnt/β-catenin pathway in the brain is associated with the development and differentiation of the nervous system, the occurrence and progression of AD, and the generation of central insulin (Kim et al. 2013; Lee et al. 2016; Wan et al. 2014). When it is activated, the stable β-catenin (np-β-catenin) increases and accumulates in the cytoplasm, and then migrates to the nucleus, up-regulating the transcription of downstream factors (Kim et al. 2013). Directly activating or inhibiting the key negative regulators of the Wnt/β-catenin pathway can alleviate the cognitive impairment in AD (Wan et al. 2014). Ex-4 can stimulate the cAMP-PKA pathway by activating the GLP-1R and then elevates the level of np-β-catenin to stimulate the Wnt/β-catenin pathway (Liu and Habener 2008). Moreover, the brain-derived insulin can be detected after the Wnt/β-catenin pathway is activated (Lee et al. 2016). This work revealed that the Wnt/β-catenin pathway was activated after the in vivo and in vitro treatment by Ex-4 based on the growing insulin levels, activated PI3K/AKT/GSK-3β signaling, and reduced tau phosphorylation. Furthermore, DKK1 was applied to block the activation of the Wnt/β-catenin signaling to illustrate the importance of this pathway for the effects of Ex-4. It was found that the effects of Ex-4 were significantly weakened or even abolished by DKK1, while it still worked in the Ex-4 group (Figs. 2, 3, 6). The results of NORT and MWMT also proved the alleviating effect of Ex-4 on the cognitive and memory impairment in T2D mice, and such alleviation was ineffective after addition of DKK1 which blocked the activation of the Wnt/β-catenin signaling (Fig. 4). All these suggest that activation of the Wnt/β-catenin signaling is the key to promote the brain-derived insulin and act the effects of Ex-4.

Moreover, mechanism by which Ex-4 increases the brain-derived insulin after activation of the Wnt/β-catenin pathway was investigated. NeuroD1, the target factor of the Wnt/β-catenin pathway, is also a transcriptional regulator of insulin. Ex-4 has been reported to significantly increase the NeuroD1 in embryonic stem cells and induce their transformation into insulin-producing cells (Zhao et al. 2016). Wnt3a can activate the Wnt pathway to upregulate the NeuroD1, increasing the transcription of *Ins2* and the generation of brain-derived insulin in the hypothalamus (Lee et al. 2016). This suggests that the Wnt/β-catenin pathway and NeuroD1 may be crucial for Ex-4 to elevate the brain-derived insulin. Thus, the in vitro and in vivo experiments were performed and disclosed that the NeuroD1 was significantly reduced in T2D models, which could be partly reversed by Ex-4 (Fig. 7). Meanwhile, the analysis on NeuroD1-overexpressing and NeuroD1-knockdown HT22 cells showed the necessity of NeuroD1 in which Ex-4 promoted the secretion of *Ins2*-derived insulin. Finally, the binding of NeuroD1 on the promoter region of the *Ins2* affected by Ex-4 could increase the brain-derived insulin in T2D (Fig. 7).

Although this work revealed the essential discoveries, it was subjected to several limitations. First, experiments were only performed on male mice due to the slight sex difference in T2D and the difficult control factors of hormonal changes in female mice. In fact, the gender metabolic differences do exist and should be studied to verify the conclusion in this work. Second, it has been reported that there appear to be two types of diabetic encephalopathy. The primary diabetic encephalopathy is a consequence of hyperglycaemia and impaired insulin action, progressing in a diabetes duration-related manner, and is linked with apoptotic neuronal loss and cognitive decline. The Secondary diabetic encephalopathy seems to arise from hypoxic-ischemic insults due to underlying microvascular disease or as a consequence of hypoglycaemia (Sima et al. 2004; Surkova 2016). While the animal model which we selected for our research, and the distinctive damage characteristics in the modeling process, are more aligned with the primary form of encephalopathy. Therefore, our study may be more meaningful and representative for the primary diabetic encephalopathy but lack the exploration and research on the hypoglycemia induced DE. Third, many brain areas, including the hypothalamus and limbic system, could synthesize the insulin, while this work focused on the hippocampus only. Whether the Ex-4 acts in other brain parts to promote the insulin and whether it can synergistically alleviate the AD-like changes in T2D need to be explored. Fourth, the regulatory process in life is usually complex and involves in multiple pathways and factors, so the Wnt/β-catenin pathway analysed in this work may not be the only pathway. In addition, the interaction of the pathway and multiple pathways was not investigated for the time being, and more detection methods are needed to discuss it in deep and detailed. In addition, although the binding of NeuroD1 on the promoter region of *Ins2* affected by Ex-4 increased the brain-derived insulin in T2D, it is still unclear if there is a multi-factor synergistic regulation in this process. Last, although clinical research also supports that Ex-4 alleviates the cognitive abnormalities in diabetic patients Gorman et al. (1988), it is a large gap to be applied clinically. All dosing methods can be repeated in human without injury, but it is impossible to obtain the brain tissue for related research. By referring to relevant studies, the measure in clinical trials need to be adapted. Specifically, p-tau181, p-tau217, and p-tau231 were detected by collecting plasma and CSF to describe the development of cognitive impairment and even AD (Janelidze et al. 2020; Ossenkoppele et al. 2022; Palmqvist et al. 2020; Thijssen et al. 2021); the levels of insulin and c-peptide in plasma and CSF would be measured to characterize the central and peripheral insulin promoted by Ex-4; and cognitive and mental state of the subjects would be measured by a scale, and cognitive decline/impairment was sub-categorized according to the diagnostic criteria (Beeri et al. 2014). In addition, intranasal drip of specific activator or inhibitor for the Wnt/β-catenin signal may confirm its importance to effects of Ex-4. Therefore, it will be further optimized and actively promoted in the follow-up studies.

## Conclusion

**Fig. 8:**
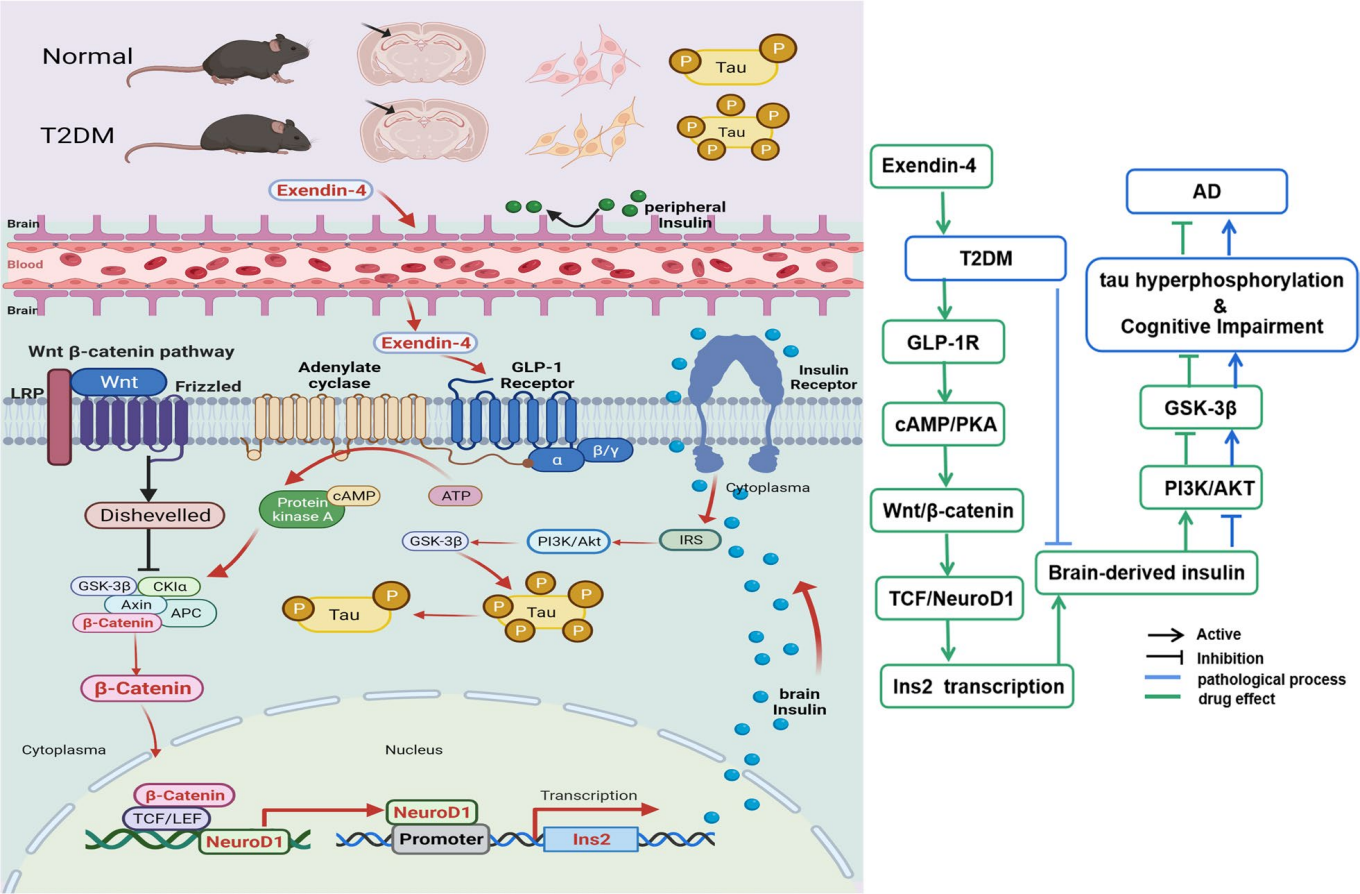
The hypothesized mechanism by which Ex-4 reduces tau hyperphosphorylation via Wnt/β-catenin/NeuroD1 signalling in T2D models. The AD-associated tau hyperphosphorylation in the hippocampus of db/db mice, HF-diabetic mice, and HT22 cells damaged by the high glucose environment was significantly higher than that in normal control. The mechanism in the current study could describe as exendin-4 activated the GLP-1 receptor on the hippocampal membrane, leading to the activation of the cAMP-PKA pathway in the GLP-1 signalling pathway, and PKA could lead to an increase in the level of nonphosphorylated β-catenin, which activated the Wnt/β-catenin signalling. Then, the downstream factor NeuroD1 of this pathway is increased and translocated into the nucleus. NeuroD1 bound to the promoter region of the insulin-encoding gene *Ins2*, thereby promoting the production and secretion of insulin. The elevated insulin activates the insulin signalling pathway and inhibits the activity of GSK-3β, ultimately reducing the hyperphosphorylation of AD-associated tau proteins regulated by GSK-3β

In summary, we described that Ex-4 ameliorated tau AD-associated hyperphosphorylation in the hippocampus in T2D. It was the Ex-4 upregulated its downstream factor NeuroD1 by activating the Wnt/β-catenin pathway in the hippocampus and promoted NeuroD1 translocated into the nucleus and binded with the promoter region of *Ins2* to increase insulin production, thus activating the insulin signalling pathway to reduce the activity of GSK-3β, which eventually leaded to a decrease in DE-associated tau hyperphosphorylation (Fig. 8). This work can not only advances our understanding of the Ex-4 effect on the DE in T2D, it also adds a new dimension in applying the Wnt/β-catenin pathway, transcription factor NeuroD1, and the *Ins2* gene as new intervention targets to the therapeutic exploration of DE brain changes in T2D.

## Supplementary Information

**Additional file 1: Table S1.** Antibodies for Western blot, IHC, IF and ChIP. **Table S2.** Primer sequences for Real-time PCR (5’-3’). **Table S3.** Predicted binding sites for NeuroD1 on *Ins2* promoter. **Figure S1. **The damage of HT22 cells induced by high glucose is not caused by increased osmotic. **Figure S2.** The blood glucose and body weight changes during experiments in HF-diabetic mice and db/db mice. **Figure S3.** The effect of knocking down the *Ins2* in HT22 cells. **Figure S4.** NeuroD1 played a crucial role in increasing the Ins2-induced brain-derived insulin and reduced tau hyperphosphorylation.

## Data Availability

The datasets used and/or analyzed during the current study are available from the corresponding author on reasonable request.

## Abbreviations

AD: Alzheimer's disease
AKT: Protein kinase B
ATCC: American Type Culture Collection
Aβ: β-Amyloid
BBB: Blood‒brain barrier
ChIP-qPCR: Chromatin immunoprecipitation coupled with quantitative polymerase chain reaction
CSF: Cerebrospinal fluid
CTL: Control
DE: Diabetic encephalopathy
DKK1: Dickkopf-1
ELISA: Enzyme linked immunosorbent assay
Ex-4: Exendin-4
GLP-1: Glucagon-like peptide-1
GLP-1R: Glucagon-like peptide-1 receptor
GSK-3β: Glycogen synthase kinase 3 beta
HEK: Human embryonic kidney
HFD: High-fat-diet
HF-diabetic: High-fat-diet/streptozotocin Induced diabetic
HG: High glucose
HGD: High-glucose-damaged
IF: Immunofluorescence
IgG: Immunoglobulin G
IHC: Immunohistochemistry
mRNA: Messenger ribonucleic acid
MUT: Mutant
MWMT: Morris water maze test
M ± SD: Means ± standard deviations
NC: Negative control
NCD: Normal control diet
NORT: Novel object recognition test
np-β-catenin: Nonphospho-β-catenin
PFA: Paraformaldehyde
qRT-PCR: Quantitative real-time PCR
RIPA: Radioimmunoprecipitation assay buffer
RT‒PCR: Quantitative real-time polymerase chain reaction
shRNA: Short hairpin RNA
STZ: Streptozotocin
T2D: Type 2 diabetes
WBG: Whole blood glucose
WT: Wild-type
