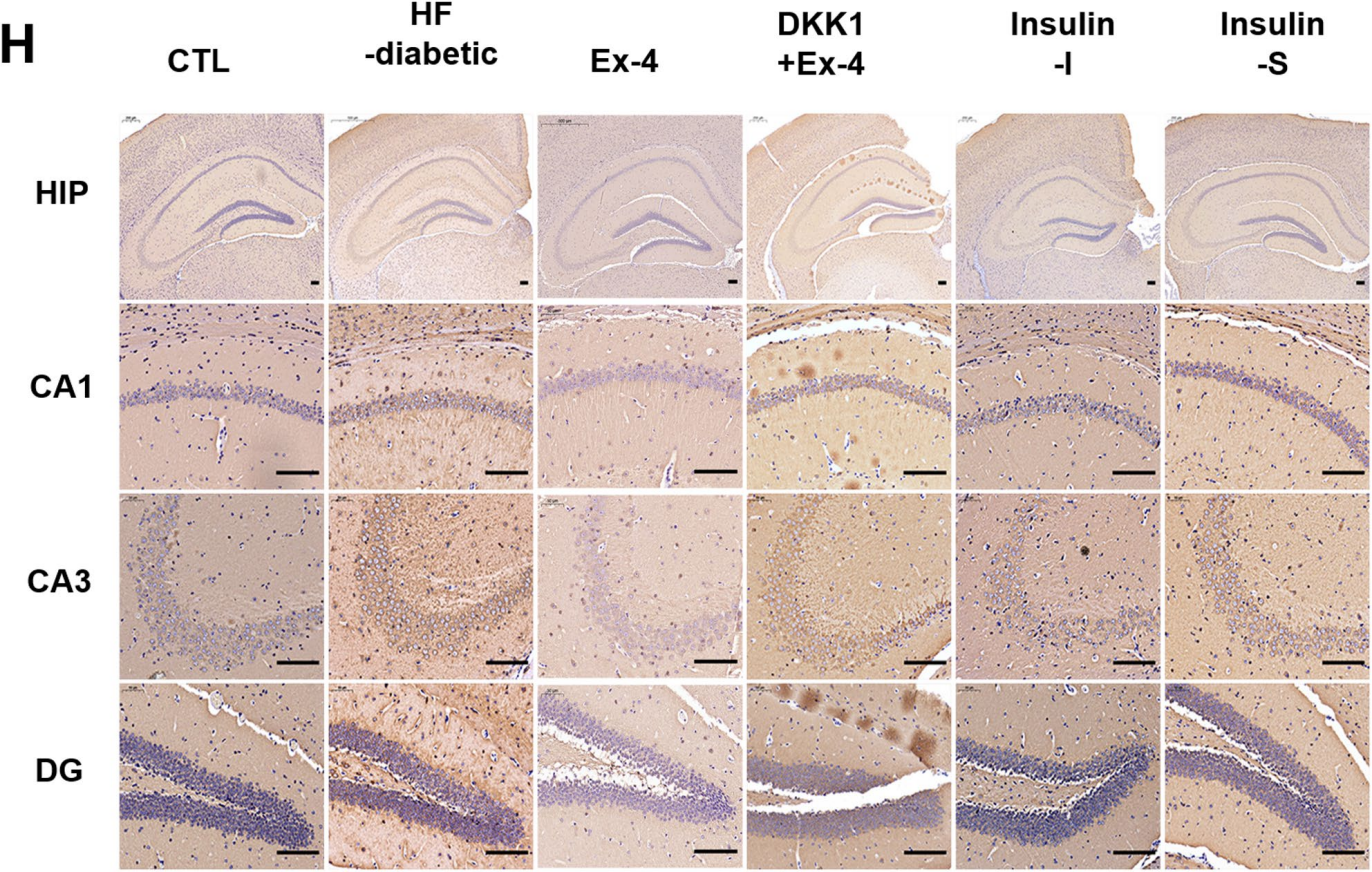# Correction: Exendin-4 ameliorates tau hyperphosphorylation and cognitive impairment in type 2 diabetes through acting on Wnt/β-catenin/NeuroD1 pathway

**DOI:** 10.1186/s10020-026-01448-x

**Published:** 2026-03-30

**Authors:** Xiaonan Kang, Dan Wang, Lu Zhang, Teng Huang, Siyue Liu, Xiaohui Feng, Yaoyao Guo, Ziyin Zhang, Zhongjing Wang, Huihui Ren, Gang Yuan

**Affiliations:** 1 Department of Endocrinology, Tongji Hospital, Tongji Medical College, Huazhong University of Science and Technology, Wuhan, 430030 China; 2 Department of Endocrinology, Tongji Medical College, The Central Hospital of Wuhan, Huazhong University of Science and Technology, Wuhan, People’s Republic of China; 3 Branch of National Clinical Research Center for Metabolic Disease, Hubei, People’s Republic of China

**Correction: Molecular Medicine 29, 118 (2023)**

**https://doi.org/10.1186/s10020-023-00718-2**

In this article (Kang et al. 2023), the wrong figure appeared as Fig. 2h. For completeness and transparency, both correct and incorrect versions are displayed below.

The original article has been corrected.

Incorrect Figure.


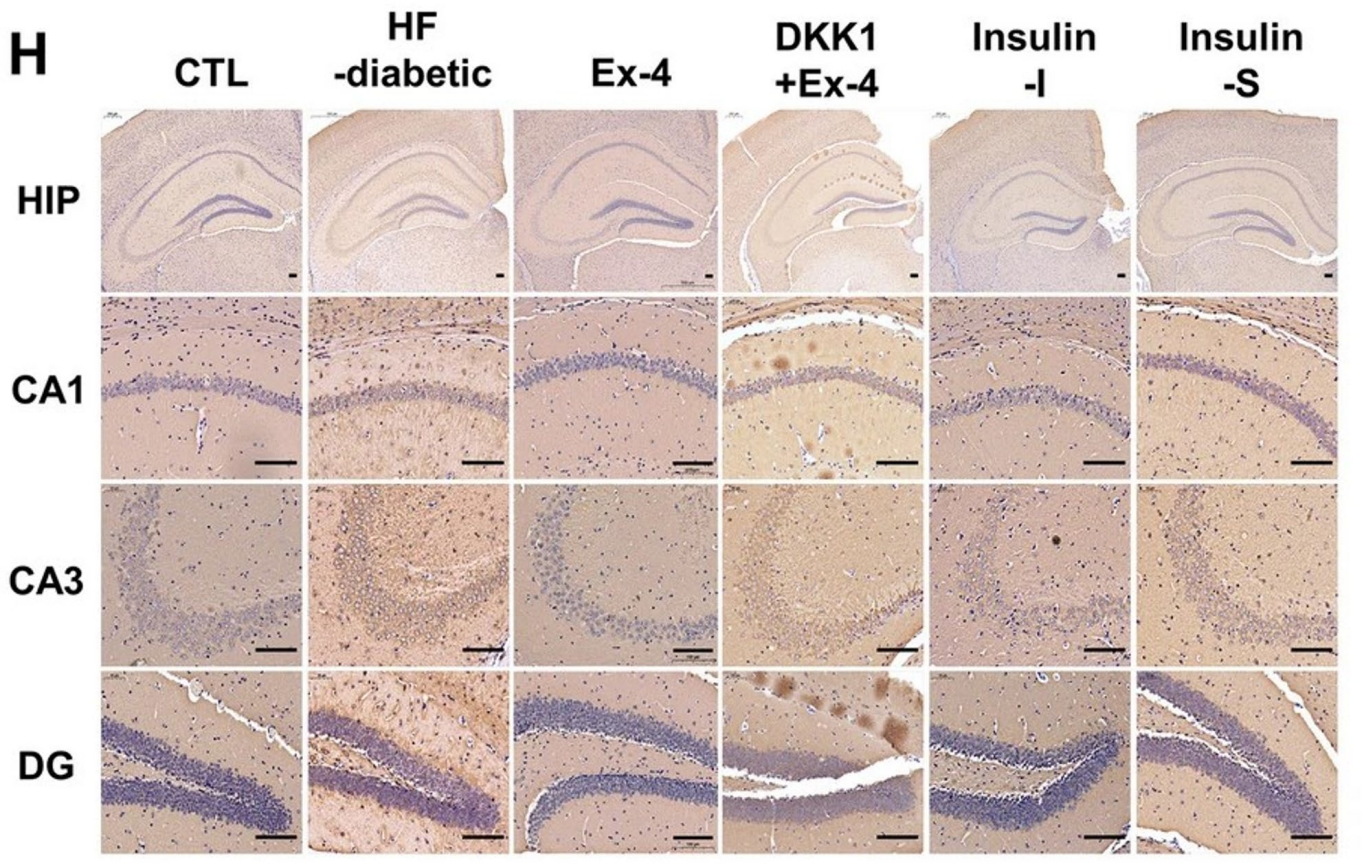


Correct Figure.