# Supplementary material for: Exendin-4 ameliorates tau hyperphosphorylation and cognitive impairment in type 2 diabetes through acting on Wnt/β-catenin/NeuroD1 pathway
Source: Mol Med. 2023 Sep 4;29:118. doi: 10.1186/s10020-023-00718-2 (PMC10478475; doi:10.1186/s10020-023-00718-2)
Supplement: Supplementary file 1 — Additional file 1: Table S1. Antibodies for Western blot, IHC, IF and ChIP. Table S2. Primer sequences for Real-time PCR (5’-3’). Table S3. Predicted binding sites for NeuroD1 on Ins2 promoter. Figure S1. The damage of HT22 cells induced by high glucose is not caused by increased osmotic. Figure S2. The blood glucose and body weight changes during experiments in HF-diabetic mice and db/db mice. Figure S3. The effect of knocking down the Ins2 in HT22 cells. Figure S4. NeuroD1 played a crucial role in increasing the Ins2-induced brain-derived insulin and reduced tau hyperphosphorylation. [file 10020_2023_718_MOESM1_ESM.doc]

**Table S1.** **Antibodies for Western blot, IHC, IF and ChIP**

| **Name** | **Source** | **Manufacturer** |
| --- | --- | --- |
| Tau (phospho S199) | Rabbit | abcam |
| Tau (phospho S202) | Rabbit | abcam |
| Tau (phospho S396) | Rabbit | abcam |
| Tau (phospho Thr217) | Rabbit | abcam |
| Tau (phospho Thr231) | Rabbit | abcam |
| Tau(D1M9X) | Rabbit | CST |
| Insulin | Mouse | CST |
| NeuroD1 | Rabbit | CST |
| Non-phospho β-Catenin (Ser33/37/Thr41) | Rabbit | CST |
| β-Catenin | Rabbit | CST |
| Phospho-Akt (Ser473) | Rabbit | CST |
| Akt | Rabbit | CST |
| Phospho-GSK-3β (Ser9) | Rabbit | CST |
| GSK-3β | Mouse | CST |
| beta Actin | Mouse | Proteintech |
| Histon H3 | Mouse | Servicebio |
| GAPDH | Mouse | Proteintech |
| Alex Fluor 488 | Goat | Proteintech |
| Alex Fluor 594 | Goat | Proteintech |
| GLP-1R | Rabbit | Proteintech |

**Table S2.** **Primer sequences for Real-time PCR (5’-3’)**

| **Gene** | **Forward** | **Reverse** |
| --- | --- | --- |
| *Ins2* | GTGACCTTCAGACCTTGGCACTG | AGGCTGGGTAGTGGTGGGTCTAG |
| *Ctnnb1* | TCGTGCTGGTGACAGGGAAGAC | ATGAAGGCGAACGGCATTCTGG |
| *Neurod1* | TGACCTTTCCCATGCTGAAT | AAGTGCTAAGGCAACGCAAT |
| *Nkx6.1* | CTGCACAGTATGGCCGAGATG | CCGGGTTATGTGAGCCCAA |
| *Isl-1* | CAGTCCCAGAGTCATCCGAGT | TGGGTTAGCAGTTTTGTCGTT |
| *Pdx-1* | GGACATCTCCCCATACGAAG | CGTTGTCCCGCTACTACGTT |
| *Mafa* | AGGAGGAGGTCATCCGACTG | CTTCTCGCTCTCCAGAATGTG |
| *Tcf7l2* | AGTCAACGCATCTATGTCTAGG | TTTTTGGAGTCCTGATGCTTTG |
| *β-actin* | CTACCTCATGAAGATCCTGACC | CACAGCTTCTCTTTGATGTCAC |
| *Glp-1r* | ACGGTGTCCCTCTCAGAGAC | ATCAAAGGTCCGGTTGCAGAA |

**Table S3. Predicted binding sites for NeuroD1 on Ins2 promoter**

| **Matrix ID** | **Name** | **Score** | **Relative score** | **Start** | **End** | **Strand** | **Predicted sequence** |
| --- | --- | --- | --- | --- | --- | --- | --- |
| MA1109.1 | NEUROD1 | 14.1454 | 0.956165375 | 1372 | 1384 | + | taacagatggaga |
| MA1109.1 | NEUROD1 | 13.5076 | 0.943992186 | 1905 | 1917 | - | cagcagatggcca |
| MA1109.1 | NEUROD1 | 11.5598 | 0.906813925 | 1382 | 1394 | + | agacagctggctt |
| MA1109.1 | NEUROD1 | 11.0914 | 0.897874181 | 435 | 447 | - | gaacagatgcctt |
| MA1109.1 | NEUROD1 | 8.54879 | 0.849342683 | 220 | 232 | - | ctccagatgttag |
| MA1109.1 | NEUROD1 | 8.4304 | 0.847083059 | 1413 | 1425 | + | ttacatatggaga |
| MA1109.1 | NEUROD1 | 8.219 | 0.843047865 | 977 | 989 | - | ggccagagggcct |
| MA1109.1 | NEUROD1 | 7.1169 | 0.82201193 | 1897 | 1909 | - | ggccagaggggct |
| MA1109.1 | NEUROD1 | 6.78402 | 0.81565817 | 1381 | 1393 | - | agccagctgtctc |
| MA1109.1 | NEUROD1 | 6.66229 | 0.813334772 | 1778 | 1790 | - | gccctgatggcct |
| MA1109.1 | NEUROD1 | 6.5616 | 0.811412922 | 1922 | 1934 | - | ctccaggtggggt |
| MA1109.1 | NEUROD1 | 6.07831 | 0.80218822 | 221 | 233 | + | taacatctggaga |


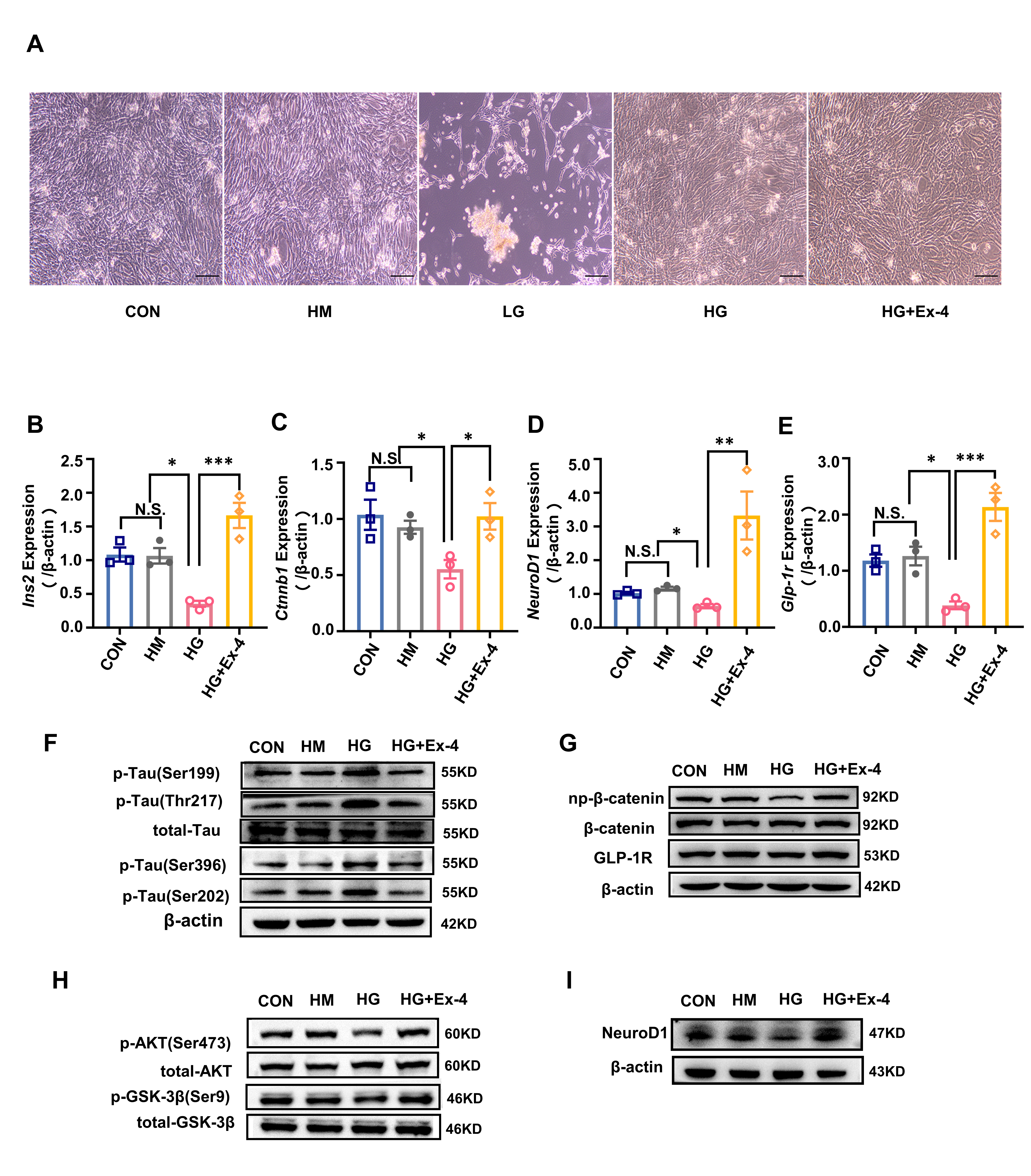


Figure S1.The damage of HT22 cells induced by high glucose is not caused by increased osmotic.

(A) the morphology changes of HT22 cells cultured in different media environments. mRNA levels of (B) *Ins2* , (C) *Ctnnb1*, (D) *NeuroD1* , (E) *Glp-1r* in groups were measured by RT-qPCR assay. β-actin was the internal control. n=3. Immunoblot demonstrated changes in (F) phosphorylated tau at Ser199, Ser202, Ser396, and Thr217 sites and total tau; (G) GLP-1R, np-β-catenin, and total β-catenin; (H) The insulin signaling activation as P-AKT^S473^ to total AKT and P-GSK-3β^S9^ to total GSK-3β; and (I) NeuroD1, β-actin was the internal control. n=3. CON: control group, DMEM (contain 25mM glucose) + 10% FBS; HM: High Mannitol group, DMEM (contain 25mM glucose + 25mM mannitol) + 10% FBS; LG: Low Glucose group, DMEM (contain 5mM glucose) + 10% FBS; HG: High Glucose group, DMEM (contain 25mM glucose + 25mM glucose) + 10% FBS; HG + Ex-4: High Glucose + Exendin-4 group, DMEM (contain 25mM glucose + 25mM glucose) + 10% FBS + 10nM Ex-4; Data are presented as the mean ± SD.*P < 0.05, **P < 0.01, *** P < 0.001.


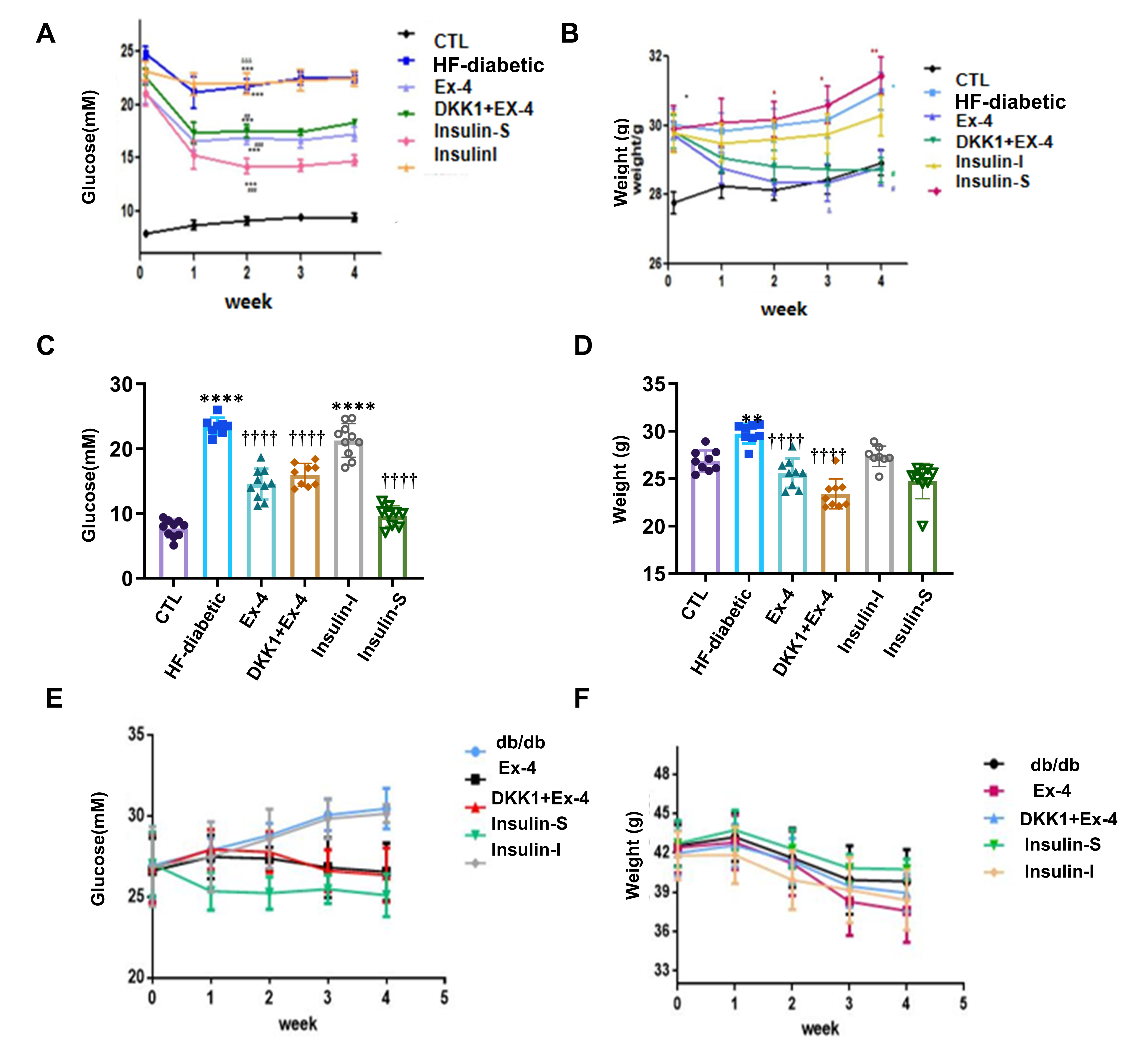


Figure S2. The blood glucose and body weight changes during experiments in HF-diabetic mice and db/db mice.

Changes in blood glucose (A) and body weight (B) of HF-diabetic mice during 4 weeks of drug intervention. Blood glucose (C) with body weight (D) at the endpoints of the study. Blood glucose (E) and body weight (F) of db/db mice changed during 4 weeks for drug intervention. Data are presented as the mean ± SD. For A-B and E-F, * P < 0.05, ** P < 0.01, ***< 0.001; For C-D, **P < 0.01, **** P < 0.0001. vs. CTL group; †††† P < 0.0001. vs. HF-diabetic group.


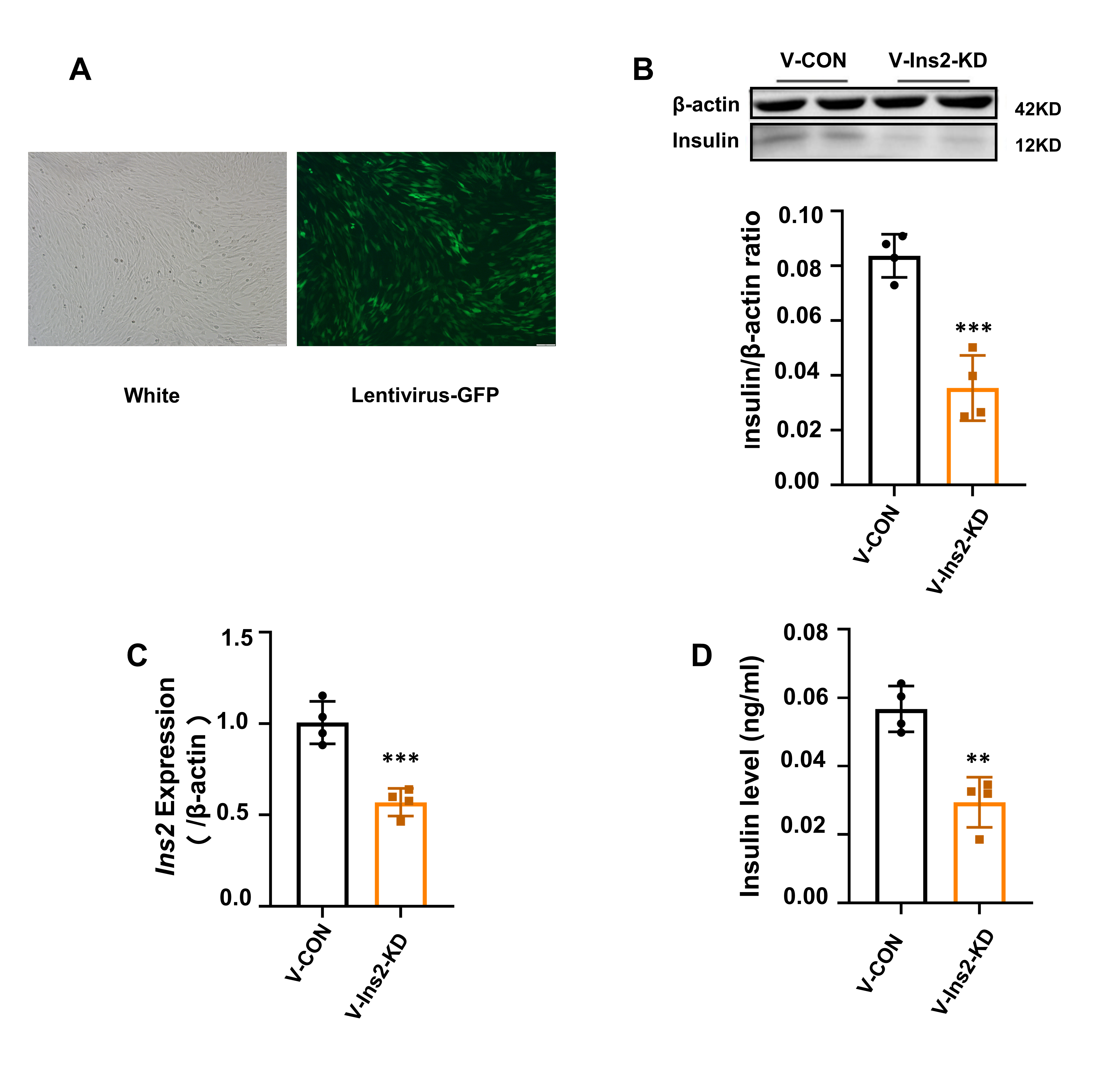


Figure S3. The effect of knocking down the *Ins2* in HT22 cells.

*Ins2* knockdown in HT22 cells by lentivirus infusion (A) was confirmed by Western blot analysis (B) and RT‒qPCR assay (C). β-actin was used as an internal standard. Insulin levels in the culture supernatant were detected by an ELISA kit (D). For A-D, n=4. Data are presented as the mean ± SD. **P < 0.01, *** P < 0.001.


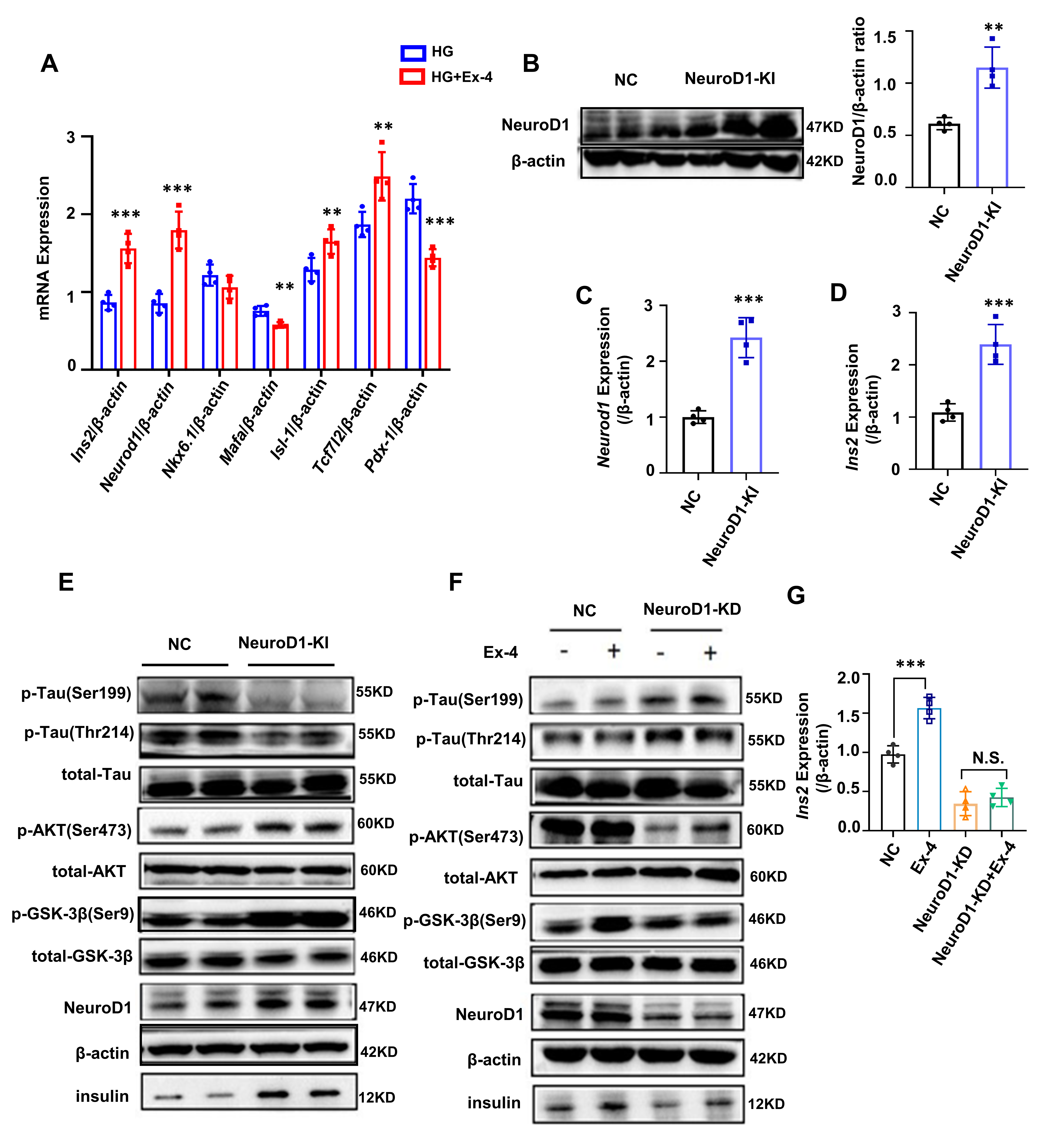


Figure S4. NeuroD1 played a crucial role in increasing the *Ins2*-induced brain-derived insulin and reduced tau hyperphosphorylation.

(A) HT22 cells were cultured in the control cultivation (CON) or high glucose (50 mM, HG) environment for 48 h, and then half of the cells were treated with exendin-4 (10 nM, HG+Ex-4) for another 48 h. The transcription regulators of insulin which have been reported, including NKX6.1, NeuroD1, Pdx-1, MAFA, and ISL-1, were measured by RT‒qPCR assay. NeuroD1 overexpression in HT22 cells by lentivirus infusion was confirmed by Western blot analysis (B) and mRNA levels of NeuroD1 (C) and Ins2 (D) were detected by RT-qPCR assay. (E) NeuroD1-overexpressing HT22 cells and negative control cells were both treated with high glucose (50 mM, HG) environment for 48 h. Then the phosphorylation levels of AD-associated tau protein, insulin and insulin signaling factors, and NeuroD1 were examined through Western blot analysis. (F-G) HT22 cells were cultured in a high glucose (50 mM, HG) environment for 48 h before infection with Ins2 knockdown or negative control lentivirus. Then, they were treated with or without Ex-4 (10 nM) for another 48 h. Then, the levels of tau phosphorylated at AD-associated sites, insulin, insulin signaling factors, and NeuroD1 were examined through Western blot analysis (F). mRNA levels of NeuroD1 in groups was detected by RT-qPCR assay (G). For A-G, β-actin is the internal control. n=4 for each group. Data are presented as the mean ± SD. ** P < 0.01, *** P < 0.001.
